# Regulating the Coordination Environment of H_2_O in Hydrogel Electrolyte for a High-Environment-Adaptable and High-Stability Flexible Zn Devices

**DOI:** 10.1007/s40820-025-01810-4

**Published:** 2025-06-12

**Authors:** Jianghe Liu, Qianxi Dang, Jodie Yuwono, Shilin Zhang, Zhixin Tai, Zaiping Guo, Yajie Liu

**Affiliations:** 1 Advanced Energy Storage Materials and Technology Research Center, Guangdong-Hong Kong Joint Laboratory for Carbon Neutrality, Jiangmen Laboratory of Carbon Science and Technology, Jiangmen, 529199 Guangdong People’s Republic of China; 2 Shenzhen Key Laboratory of Advanced Materials, School of Materials Science and Engineering, Harbin Institute of Technology, Shenzhen, 518055 Guangdong People’s Republic of China; 3 Faculty of Sciences, Engineering and Technology, School of Chemical Engineering, University of Adelaide, Adelaide, SA 5005 Australia

**Keywords:** Coordination environment of water, High environmental adaptability, Hydrogel electrolyte, Side reactions, Low-temperature performance

## Abstract

Aqueous zinc-ion batteries are promising candidates as stationary storage systems for power-grid applications due to their high safety and low cost. The practical implementation of Zn-ion batteries currently still faces formidable challenges because of Zn dendrite growth, hydrogen evolution, and inadequate environmental adaptability. Herein, to address these challenges, a strategy of regulation of water molecules coordination in electrolyte is proposed via developing a cross-linked hydrophilic hydrogel polymer electrolyte. Within this system, the continuous hydrogen bond among H_2_O molecules is disrupted and the isolated H_2_O molecules are strongly bound with a polymeric matrix comprised of polyacrylamide, carboxymethyl cellulose, and ethylene glycol, which can restrain the activity of H_2_O molecules, thus effectively alleviating Zn dendrite growth and hydrogen evolution and enhancing the anti-freezing ability. With this electrolyte, the Zn||Cu cell presents a high coulombic efficiency of 99.4% over 900 cycles and Zn||Zn symmetric cell exhibits high cycling stability, maintaining plating/stripping for over 1,700 h. Moreover, the assembled Zn||PANI device also demonstrates outstanding electrochemical performance over a wide-temperature range, including a long cycling life over 14,120 cycles at room temperature and an ultralong cycling surpassing 30,000 cycles even at − 40 °C. This showcases the manipulation of water coordination chemistry for advanced, highly adaptable batteries.

**Supplementary Information:**

The online version contains supplementary material available at 10.1007/s40820-025-01810-4.

## Highlights

- A hydrogel electrolyte regulation strategy of the water coordination environment can effectively restrain the hydrogen evolution reaction, Zn corrosion and dendrite formation in the wide-temperature ranges.
- Prepared high-environment-adaptable hydrogel electrolytes (HEA-3) exhibits a high reversibility of zinc deposition/stripping (coulombic efficiency of 99.4% for Zn||Cu cell).
- Zn-based device using HEA-3 electrolyte can do cycling over 30,000 cycles at a large current density of 2 A g^−1^ at − 40 °C and also can do normal cycling even at -70 °C.

**Supplementary Information:**

The online version contains supplementary material available at 10.1007/s40820-025-01810-4.

## Graphical abstract

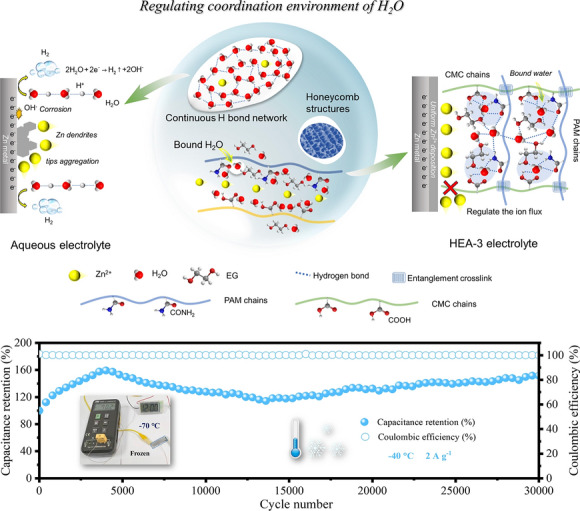

## Introduction

Nowadays, safety concerns have greatly impeded the large-scale development of lithium-ion batteries (LIBs), particularly for stationary energy storage systems [1]. This is primarily because the organic electrolytes used in conventional LIBs are highly flammable and volatile, which may exacerbate thermal runaway and eventually lead to fire and explosion when short circuits, overcharging, or other thermal abuse occurs. Rechargeable aqueous batteries, using inherently safe and cost-effective water-based electrolytes, offer a promising solution to address these concerns. Among various aqueous batteries, aqueous zinc metal batteries (AZMBs) have gained significant attention in the field of energy storage due to their low cost, high safety, and high theoretical specific capacity (5,854 mAh cm^−3^) [2–5]. However, the cutting-edge AZMBs face substantial challenges, including Zn dendrite formation during the stripping and plating processes, hydrogen evolution in liquid electrolytes, metal corrosion at the interface, and insufficient ion conductivity at low temperatures [6, 7]. These issues hinder cycling stability and restrict their application in a broad temperature range.

In AZMBs, extensive hydrogen-bond networks between water molecules significantly enhance proton (H^+^) diffusion [8–10]. As a result, the hydrogen/oxygen evolution reaction will occur upon H^+^ reaches the metal surface. According to 2H_2_O + 2e^−^ → H_2_↑ + 2OH^−^ (-0.762 V vs. SHE), the generated H_2_ can cause battery bulges, while the substantial production of OH^−^ leads to the formation of basic by-products that insulate and passivate the Zn anode surface, descending the cycling reversibility and accelerating the growth of Zn dendrites [11–14]. Moreover, the continuous hydrogen bonding between water molecules is dynamic, constantly breaking and re-forming within seconds, and this process is temperature-dependent. At low temperatures, hydrogen-bond recombination becomes dominant, triggering a phase transition from liquid to solid water. Consequently, this freezing leads to reduced ionic conductivity and increased low-temperature polarization, thereby significantly limiting the application of aqueous zinc-based energy storage devices in low-temperature environments [15].

To tackle these challenges in AZMBs, substituting the conventional aqueous electrolyte with a hydrogel electrolyte is considered as a viable and effective approach. Hydrogel systems utilizing polymers such as polyvinyl alcohol (PVA), polyethylene glycol (PEG), xanthan gum (XG), and sodium alginate (SA) disrupt hydrogen bonding networks among water molecules through their polymeric frameworks, thereby inhibiting the activity of H_2_O molecules and reducing side reactions between electrode and electrolyte. However, conventional hydrogels exhibit limited low-temperature applicability due to insufficient disruption of water hydrogen bonds. The introduction of chaotropic zinc salts (e.g., Zn(ClO_4_)_2_, Zn(CF_3_SO_3_)_2_, etc.) enhances anti-freezing capabilities by increasing the tetrahedral entropy of water molecules, which accelerates the dissociation of hydrogen bond between water molecules [16]. Consequently, chaotropic salt-containing hydrogels achieve high low-temperature ionic conductivity. Nevertheless, most hydrogel matrices (e.g., PVA, PEG, XG, SA) exhibit poor salt tolerance with high-concentration chaotropic salts, leading to gel disintegration. In contrast, polyacrylamide (PAM) demonstrates exceptional salt tolerance, enabling stable integration with concentrated chaotropic Zn salts while maintaining structural integrity [17]. As a result, the PAM-based hydrogel electrolytes deliver superior ionic conductivity over a broad temperature range, including at low temperatures, as evident in Fig. S1a [S1–S9]. Therefore, PAM is a promising matrix for the development of highly adaptable hydrogel electrolytes under various environmental conditions. However, the insufficient mechanical strength of pure PAM necessitates its combination with other components, and the challenge of balancing mechanical strength and wide-temperature ion conductivity in PAM-based composite system warrants further exploration.

To address the challenge and develop an environmentally adaptable hydrogel electrolyte for wide-temperature zinc energy storage, carboxymethyl cellulose (CMC) and ethylene glycol (EG) were incorporated into polyacrylamide (PAM) to create a cross-linked hydrophilic hydrogel polymer electrolyte. CMC chains can interact with PAM chains to form a semi-interpenetrating network through strong hydrogen bonding between their molecular chains and physical entanglement of the chain segments, thereby enhancing mechanical strength compared to other PAM-based hydrogel systems (Fig. S1b) [S9–S13]. More importantly, the CMC polymer chains and EG molecules provide abundant H bond acceptor and donor groups, such as –COOH and –OH, which can bind with H_2_O molecules, further regulating the coordination environment of H_2_O in PAM-based hydrogel electrolyte, thus restraining activity of H_2_O molecules and enhancing the anti-freezing ability while maintaining high ion conductivity. Consequently, the Zn||Cu asymmetric cell using the prepared HEA-3 electrolyte achieves a high coulombic efficiency of 99.4% after 900 cycles. Besides, the HEA-3 electrolyte also demonstrates a high ionic conductivity of 4.12 × 10^–3^ S cm^−1^ even at -50 °C, enabling the assembled Zn||Zn cell to cycle for over 1400 h at a current density of 1 mA cm^−2^, even at − 40 °C. Furthermore, owning to the excellent mechanical properties and anti-freezing ability of the fabricated HEA-3 electrolyte, the flexible Zn||PANI device using this hydrogel polymer electrolyte demonstrates superior cycling stability (over 30,000 cycles at − 40 °C). This indicates that flexible devices hold great promise for future wearable applications, particularly in extreme conditions.

## Experimental and Calculation

### Preparation of Electrolytes and Electrodes

#### Fabrication of Electrolytes

The high-environment-adaptable (HEA) hydrogel electrolytes were prepared by free radical polymerization. First, 5 mg ammonium persulfate (APS, purity ~ 99.99%, Aladdin) initiator and 1 mg N, N′-methylene bisacrylamide (MBAA, purity ~ 98%, Aladdin) cross-linker were added to 5 mL different concentrations (1, 2, 3, and 4 M marked as HEA-1, HEA-2, HEA-3, and HEA-4, respectively) of Zn(ClO_4_)_2_•6H_2_O (purity ~ 98%, Aladdin) aqueous electrolyte at room temperature and stirred for 2 h. Whereafter, 1 g polyacrylamide (AM, purity ~ 98%, Aladdin), 0.05 g carboxymethyl cellulose (CMC, M.W. 250,000, Aladdin), and 1.25 g ethylene glycol (EG, purity ~ 99%, Aladdin) were added into the above solution and continuously stirred for 5 h to obtain a homogeneous and transparent precursor solution (Fig. S2a). The homogeneous precursor solution is poured into glass Petri dishes and sealed with cling film. After that, these Petri dishes were placed in a vacuum oven with a temperature of 60 °C for 8 h to obtain the transparent and stretchable hydrogel electrolyte membrane (Fig. S2b). In addition, the other hydrogels without Zn salts are named PAM, PAM/CMC, and PAM/CMC/EG hydrogels, respectively. The Zn(ClO_4_)_2_ + EG aqueous electrolyte prepared through 1.25 g EG incorporates into the 3 M Zn(ClO_4_)_2_•6H_2_O aqueous electrolyte.

#### Fabrication of Flexible Polyaniline Cathode

The cyclic voltammetry (CV) method was used to electrodeposit PANI (AR, Aladdin) on the carbon cloth in the 0.2 M aniline monomer/0.5 M H_2_SO_4_ solution; the platinum foil and saturated calomel electrode (SCE) served as the counter electrode and reference electrode, respectively. The scan ranges from − 0.2 to 1 V with a scan rate of 50 mV s^−1^; the loading amount of polyaniline was about 1 mg cm^−2^.

#### Fabrication of Flexible Zn Electrode

The carbon cloth served as the working electrode, the 1 M ZnSO_4_ aqueous solution as the electrolyte, and the Zn metal as the counter and reference electrode. The flexible Zn anode was prepared via electroplating Zn into the carbon cloth under an electroplating voltage of − 0.8 V.

### Structural Characterization

The structures of the electrolytes were analyzed by the Fourier transform infrared (FTIR) spectra using a Nicolet iS50 FTIR spectrometer in the range of 650–4000 cm^−1^. The morphologies of the hydrogel electrolyte and the Zn anodes were observed by the Hitachi S4700 scanning electron microscope (SEM). The anti-freezing property of the samples was investigated by the differential scanning calorimetry (DSC, Mettler-Toledo DSC3). The testing temperature ranges from 20 to − 65 °C with a cooling rate of 5 °C min^−1^ rate in a N_2_ atmosphere. The thermal stability of the samples was evaluated using a thermal analyzer (Netzsch STA449F5) with a range of 25 − 600 °C at 10 °C min^−1^ under N_2_ atmosphere. The mechanical properties of the samples (50 × 10 × 1 mm^3^) were measured using a universal testing machine (MTS, CMT6104) with a tensile rate of 10 mm min^−1^. The X-ray diffraction (XRD) patterns of the Zn anodes were recorded using a PHI 5000 VersaProbe II with Al Kα irradiation (1486.6 eV).

### Electrochemical Test

The bulk resistance of hydrogel electrolytes was evaluated by AC impedance spectroscopy. The hydrogel electrolytes were placed between two pieces of stainless-steel electrodes, and the frequency ranges from 0.01 to 1 MHz with an amplitude of 10 mV. The Zn^2+^ transference number of the hydrogel electrolyte was investigated by the combined direct-current polarization method with AC impedance spectroscopy. The symmetric Zn||Zn cell with hydrogel electrolyte was polarized using a 10-mV polarization voltage for 1 h. AC impedance spectroscopy was applied to obtain the relevant resistance values before and after polarization. The hydrogen evolution reaction (HER) and oxygen evolution reaction (OER) were investigated by linear sweep voltammetry (LSV) method in the Zn||Ti cell; the working voltage ranges from − 0.4 to 3 V with a scan rate of 1 mV s^−1^. The nucleation behavior of Zn was evaluated by the GCD technique in the Zn|| Ti cell with a constant current density of 1 mA cm^−2^. The corrosion behavior was evaluated using Tafel tests in the Zn||Zn cell; the voltage ranges from − 0.15 to 0.15 V with a scan rate of 1 mV s^−1^. Hydrogen evolution in cell was quantified via an in situ differential electrochemical mass spectrometry (DEMS; HPR-20EGA) using a sealed Zn||Zn cell model cycled at 2 mA cm⁻^2^/1 mAh cm⁻^2^ (Ar as the carrier gas). Real-time gas analysis confirmed H₂ generation dynamics in the cell. The Zn plating behavior in different electrolytes is observed by an in situ optical microscopy at a current density of 5 mA cm⁻^2^. The long-term plating and stripping behavior of Zn were measured by the Neware battery test system with Zn||Zn cell and Zn||Cu cell. The electrochemical performance of the Zn||PANI devices was also evaluated by the CV and GCD techniques.

### Density Functional Theory Calculations

Density functional theory (DFT) calculations were performed using the projector augmented wave (PAW) method [18, 19] as implemented in the Vienna Ab initio Simulation Package (VASP) [20, 21]. The calculations were completed with a plane-wave cutoff energy of 500 eV and a single Gamma k-point. The electronic self-consistent calculation was converged to 1 × 10^–5^ eV, and ionic relaxation steps were performed using the conjugate-gradient method (IBRION = 2) and continued until the total force on each atom dropped below a tolerance of 1 × 10^–2^ eV Å^−1^. The generalized gradient approximation (GGA) was used for the exchange correlation functionals as parameterized by Perdew–Burke–Ernzerhof (PBE) [22]. The dispersion correction was also included in this study by using the DFT D-3 method [23]. Binding energy (*E*_BE_) between two different molecules (X and Y) is calculated using the following equation:

1 $$E_{{{\text{BE}}}} = E_{{{\text{XY}}}} {-}E_{X} {-} \, E_{Y}$$

where *E*_XY_, *E*_*X*_, and *E*_*Y*_ are the total electronic energies of molecules *X* and *Y*, molecule *X* and molecule *Y*, respectively. The interaction between molecules considered is water–water, water–ethylene glycol, water–carboxy methyl cellulose monomer, and water–acrylamide monomer unit.

### Molecular Dynamics (MD) Simulations

All MD simulations were performed using the Generalized Amber Force Field (GAFF) [24]. The ACPYPE was employed to obtain the force field topology [25]. The simulation box size of 45 × 45 × 45 Å^3^ was used in all simulation models. The simulation systems consist of Zn^2+^, ClO_4_^−^, H_2_O, with the absence and presence of EG, CMC, and AM molecules to model the so-called aqueous electrolyte and hydrogel electrolyte. The ratio of the gel electrolyte component in its monomer form is as follows: Zn(ClO_4_)_2_:H_2_O:EG:CMC:AM = 1:24.52:1.34:0.01:0.94. The cutoff distance of 1.2 nm was used for Lennard–Jones potential. The Coulombic potential was measured using Particle Mesh Ewald (PME) with a cutoff distance of 1.2 nm and Fourier grid spacing of 0.12. All bonds were constrained with the LINCS algorithm. Periodic boundary conditions were applied in all directions. The MD simulations were started by running initial energy minimization, followed by 1,000 ps of NVT simulation and 1,000 ps of NPT simulation with an integration time step of 0.001 ps. All the simulation systems were finally maintained at 298 K using the Nose–Hoover thermostat for 40 ns to collect simulation data. A time constant of 1 ps was applied for the temperature coupling.

### Calculation Formula

Ionic conductivity calculation:

2 $$\sigma = \frac{l}{{R_{{\text{b}}} S}}$$

where *l* represents the thickness of the hydrogel electrolyte, *R*_b_ is the bulk resistance of the hydrogel electrolyte, and *S* is the contact area between electrolyte and electrode.

Zn^2+^ transfer number calculation:

3 $$t_{{{\text{Zn}}^{2 + } }} = \frac{{I_{{\text{S}}} (\Delta V - I_{0} R_{0} )}}{{I_{0} (\Delta V - I_{{\text{S}}} R_{{\text{S}}} )}}$$

Zn^2+^ ionic conductivity calculation:

4 $$\sigma_{{{\text{Zn}}^{2 + } }} = \sigma_{{\text{T}}} \times t_{{{\text{Zn}}^{2 + } }}$$

where Δ*V* is polarization voltage,* I*_0_ represents the initial current, *I*_s_ is the steady current, *R*_0_ is the initial interfacial resistance, *R*_s_ represents the steady interfacial resistance, and the σ_T_ is the total ionic conductivity.

The formula used to calculate the depth of discharge (DOD) for a Zn metal anode using Zn foil is as follows [26]:

5 $${\text{DOD}} = \frac{y}{{C_{{{\text{Zn}},{\text{volume}}}} \times 10^{ - 4} }} \times 100\% = \frac{y}{0.585x} \times 100\%$$

where* x* (μm) represents the thickness of the Zn electrode and *y* (mAh cm^−2^) is the Zn charge–discharge areal capacity, *C*_Zn, volume_ is the theoretical volume capacity.

In general, the relationship between the nucleation overpotential (*η*) and critical Zn nuclei radius (*r*) follows the formula [27, 28]:

6 $$r = 2\frac{{\gamma V_{{\text{m}}} }}{F\left| \eta \right|}$$

where *γ* represents the surface energy of the interface between Zn and electrolyte, *V*_m_ stands for the molar volume of Zn, *F* marks Faraday's constant, and *η* represents the nucleation overpotential.

7 $$i = av^{b}$$

or

8 $$\log (i) = b\log (v) + \log (a)$$

where *i* represents the response currents, *v* is the scan rates. In general, the electrochemical reaction is controlled by the diffusion process if the parameter *b* = 0.5. Otherwise, the electrochemical reaction is controlled by the capacitive process if the parameter *b* = 1. For a specific voltage, the *b* values are obtained by the slope of the log(*v*)–log(*i*) plots.

Capacitive contribution analysis:

9 $$i = k_{1} v + k_{2} v^{1/2}$$

or

10 $$i/v^{1/2} = k_{1} v^{1/2} + k_{2}$$

where *k*_1_ and *k*_2_ are surface capacitance and diffusion capacitance contribution constant, respectively.

## Results and Discussion

### Working Mechanism of Regulating Coordination Environment of Water Molecules

**Fig. 1:**
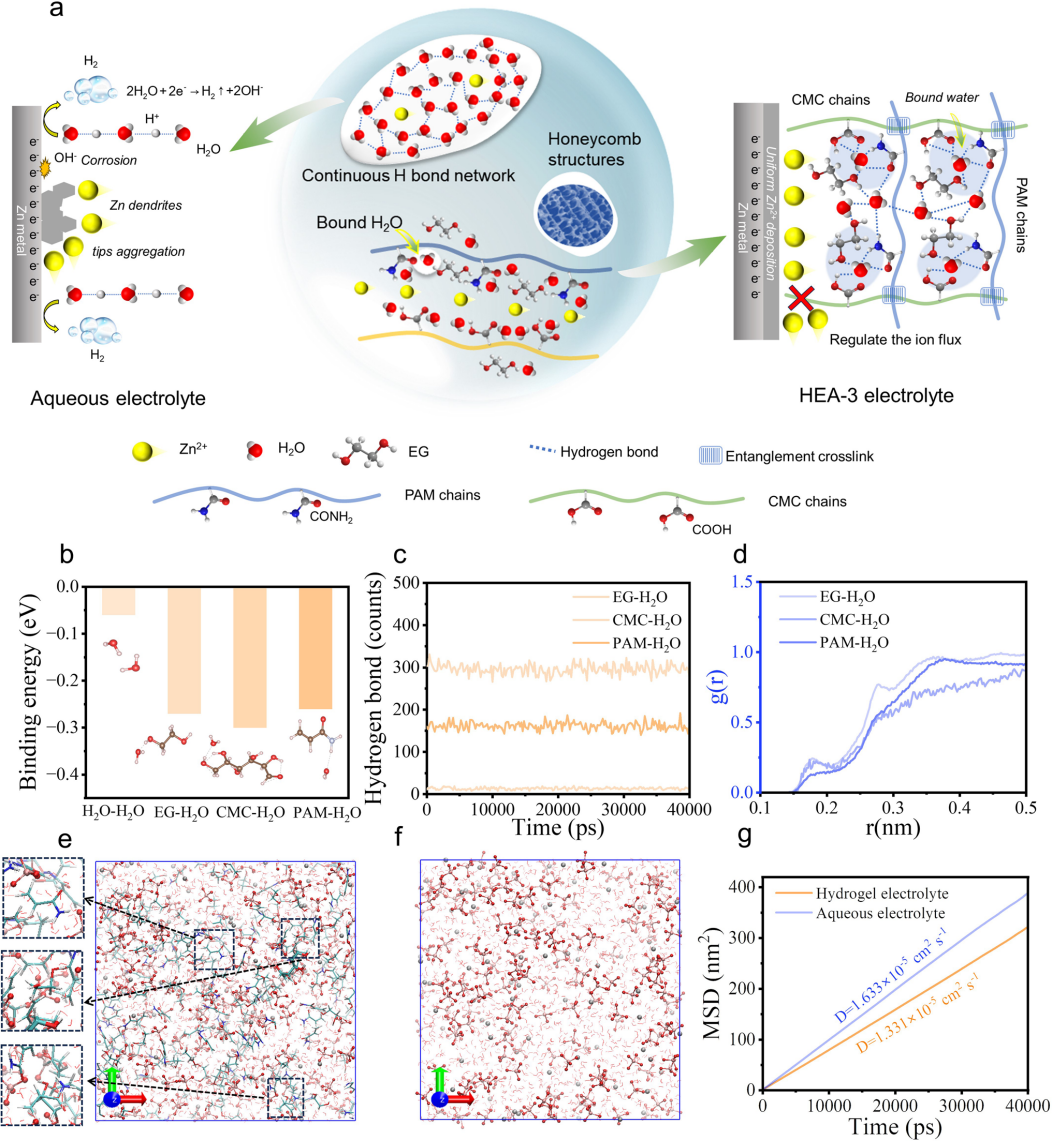
**a** Schematic illustration of the regulation effect of the coordination environment of water molecules in HEA-3 electrolyte. **b** Binding energy between EG, CMC, and PAM with H_2_O, respectively. **c** Counts of the hydrogen bonds formed between water and various components in the HEA-3 electrolyte. **d** Radial distribution functions (RDF) of EG-H_2_O, CMC-H_2_O, and PAM-H_2_O. Snapshot of the local structures of the **e** HEA-3 electrolyte and **f** aqueous electrolyte using MD simulations. **g** Simulated MSD versus time curves for water molecules in different electrolytes

In AZMBs, owing to the significant electronegativity difference between O and H atoms, hydrogen bonds readily form among water molecules, promoting a continuous hydrogen-bond network in the aqueous electrolyte. The continuous hydrogen-bond network enhances rapid proton (H^+^) transportation, leading to the occurrence of HER when H^+^ reaches the Zn metal surface. Concurrent hydroxide ion (OH⁻) generation corrodes the Zn metal surface, resulting in the formation of non-uniform insulating by-products, such as Zn_4_ClO_4_(OH)_7_ and Zn_5_(OH)_8_Cl_2_. These insulating by-products on the surface of Zn anode deteriorate Zn^2+^ plating behavior, ultimately causing Zn dendrite growth [11–14]. Here, a cross-linked hydrophilic hydrogel polymer electrolyte was developed to optimize water molecule distribution and disrupt the hydrogen-bond network, effectively alleviating the aforementioned issues. Within this system, the PAM, CMC polymer chains and EG molecules provide abundant H bond acceptor and donor groups (–CONH_2_, –COOH, –OH), dynamically coordinating water molecules to regulate the coordination environment of H_2_O. This process restrains the electrochemical activity of water molecules and hinders the proton (H^+^) rapid diffusion, thus effectively suppressing HER and other side reactions (Fig. 1a).

DFT and MD simulations provide deep insights into the coordination environment of water molecules within the hydrogel. The binding energy of H_2_O-EG, CMC-H_2_O, and PAM-H_2_O is − 0.27, − 0.30, and − 0.26 eV, respectively (Fig. 1b). These values are much higher than that of H_2_O-H_2_O (-0.06 eV), indicating that the H_2_O molecule prefers to bond with EG, CMC, and PAM rather than with other H_2_O molecules. Therefore, the H_2_O can be redistributed, allowing more hydrogen bonds (HBs) between EG, polymer molecular chains, and H_2_O that could be formed in this prepared HEA-3 electrolyte (Fig. 1c). Meanwhile, it also indicates that the number of HBs in the hydrogel system remains constant over time, suggesting that the reconstructed multiple hydrogen bonds network in polymeric HEA-3 electrolyte is stable. The radial distribution function (RDF) results indicate that the coordination environment of H_2_O molecules in the HEA-3 electrolyte undergoes changes due to the reconstructed multiple hydrogen bonds network formed (Fig. 1d). In addition, MD simulations have further confirmed the differences in the coordination environment of H_2_O molecules in various electrolytes. In the HEA-3 electrolyte system, the continuous HB network among H_2_O molecules is disrupted and redistributed, with a higher proportion of H_2_O primarily coordinated by non-aqueous molecules in this restructured multiple hydrogen-bond network (Fig. 1e). In contrast, a continuous and uniform hydrogen bond network exists between H_2_O molecules in the aqueous electrolyte, enhancing rapid proton (H^+^) transport and potentially leading to significant side reactions (Fig. 1f). Besides, the self-diffusion coefficient of H_2_O, derived from mean square displacement (MSD) curves in HEA-3 electrolyte, is 1.331 × 10^–5^ cm^2^ s^−1^, which is lower than that in aqueous electrolyte (1.633 × 10^–5^ cm^2^ s^−1^) (Fig. 1g). Due to interruption of HBs among water molecules in HEA-3 electrolyte, their proton (H^+^) transportation behavior is effectively inhibited.

### Preparation and Characterization of High-Environment-Adaptable Hydrogel Electrolytes

**Fig. 2:**
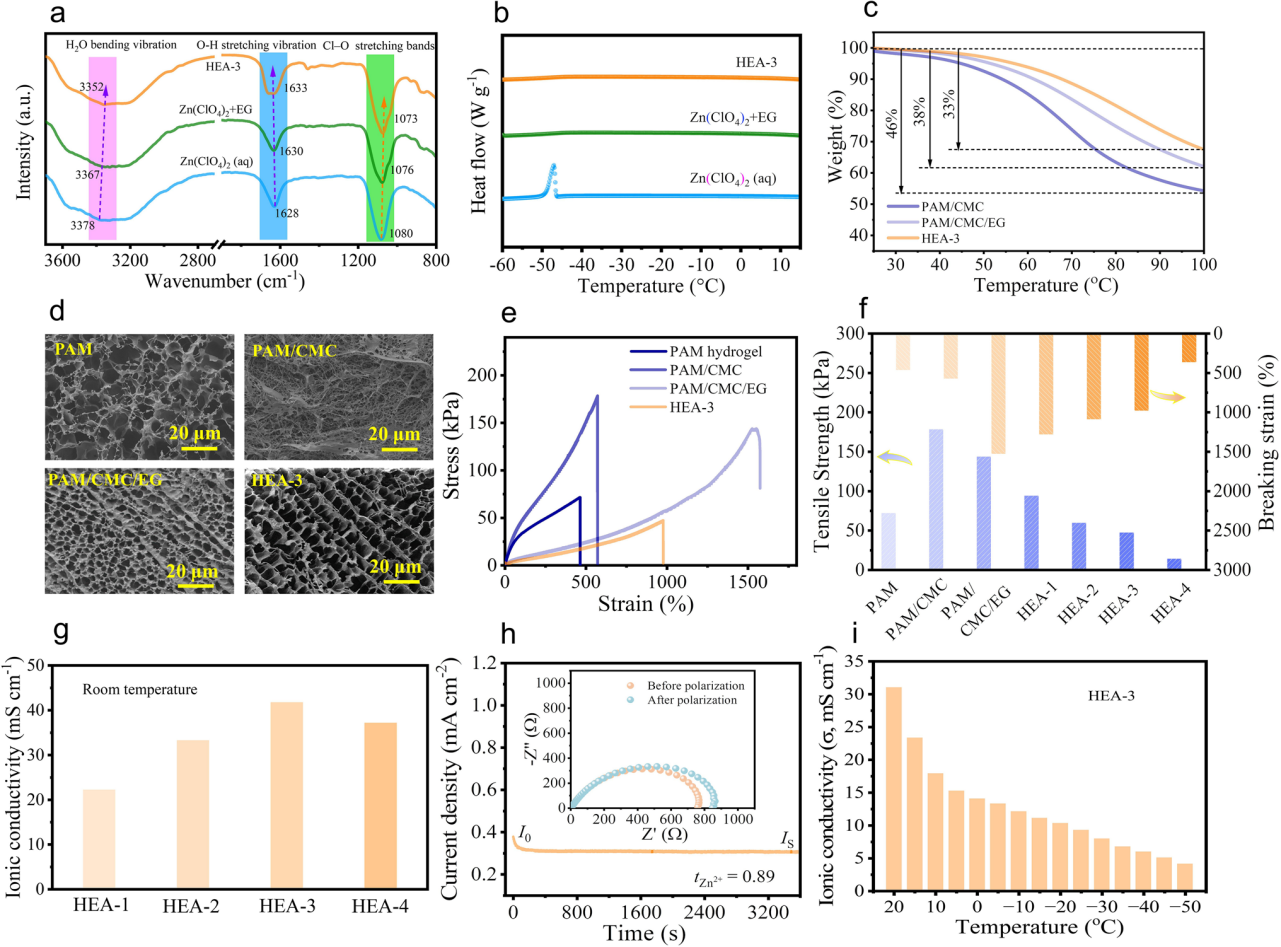
**a** FTIR spectrum of the Zn(ClO_4_)_2_ (aq), Zn(ClO_4_)_2_^+^EG, and high-environment-adaptable (HEA)-3 electrolyte and **b** their DSC curves. **c** TGA curves of the PAM/CMC hydrogel, PAM/CMC/EG hydrogel, and HEA-3 electrolytes. **d** SEM images of hydrogel electrolytes. **e** Strain–stress curves of the hydrogel electrolytes, and **f** corresponding mechanical properties. **g** Room-temperature ionic conductivities of hydrogel electrolytes. **h** DC polarization curve of the cell with HEA-3 electrolyte at room temperature (the inset shows the AC impedance curves of the Zn|HEA-3|Zn symmetric cell before and after polarization). **i** Ionic conductivity of HEA-3 electrolyte at different temperatures

The HEA-3 electrolytes are prepared via the free radical polymerization method, where PAM serves as the host polymer and combines with CMC and EG to form a semi-interpenetrating polymer network. The HEA-3 electrolyte is a flexible, transparent and stretchable membrane, as shown in Fig. S2. The FTIR spectrum of various electrolytes is depicted in Fig. 2a. In the Zn(ClO_4_)_2_ aqueous electrolyte, distinct peaks at 1080, 1628, and 3378 cm^−1^ correspond to the Cl-O stretching vibration, O–H stretching vibration, and H_2_O bending vibration, respectively [29, 30]. Upon the introduction of EG, the H_2_O bending vibration undergoes a redshift, while the O–H stretching vibration suffers a blue shift. This indicates the formation of more HBs due to the high intermolecular electrostatic interactions between EG and H_2_O. When polymer (PAM/CMC) is incorporated into hydrogel electrolyte, similar peak shifts as observed with EG effect further suggest that HBs are formed between the polar functional group of the polymer chains and H_2_O molecules, disturbing the HB network among H_2_O molecules [31].

In addition, the FTIR spectra offer more structure details for polymeric matrix in electrolytes. As shown in Fig. S3, the distinct peaks at 1454, 1612, 1659, and 3202 ~ 3350 cm^−1^ correspond to the stretching vibration of the C-N bond, the bending vibration of the N–H bond, the stretching vibration of the C=O and O–H bond, and the vibration of O–H and N–H bond, respectively [32–34]. Upon the introduction of CMC, new peaks at 1325, 1419, and 1587 cm^−1^ in the spectra of PAM/CMC hydrogels are attributed to the symmetric and asymmetric stretching vibrations of COO- derived from the CMC, indicating that the CMC is completely integrated into the hydrogel network. Furthermore, compared to pure PAM hydrogels, the peak of the C=O bond and O–H bond in PAM/CMC gel appears around at 1635 cm^−1^, while the N–H bond and O–H bond peaks are in the range of 3236 ~ 3340 cm^−1^, suggesting the formation of a physical cross-linked semi-interpenetrating network between PAM and CMC through hydrogen bonding [33, 34]. Furthermore, the peak as associated with C=O and O–H bond blueshift to 1662 cm^−1^, evidencing that the hydrogen bonding is enhanced with the introduction of EG in the PAM/CMC/EG system. These results further signify that a stable reconstructed multiple hydrogen bonds network is formed in the HEA hydrogel. Besides, as the contents of Zn(ClO_4_)_2_ increase from HEA-1 to HEA-4 electrolyte, the peak intensities located at 1662, 1419, and 1038 cm^−1^ decreases, and the peak around 1082 cm^−1^ shifts to 1072 cm^−1^, and the peak around 1612 cm^−1^ shifts to 1628 cm^−1^, suggesting interaction among Zn^2+^, ClO_4_^−^, EG molecules, and polymer chains. The Zn^2+^ and ClO_4_^−^ can dissociate the interactions between the polymer molecular chains and encourage more water molecules to be captured by the polymer molecular chains [35]. These interactions will significantly restrain the amounts of free water in the hydrogels, which will be beneficial for the suppressing side reaction and dendrite formation during charge/discharge cycling. Since water freeze is mainly attributed to the interaction of HBs among H_2_O molecules, the freeze point can be decreased via abating the HB interaction of H_2_O–H_2_O [35–37]. As shown in Fig. 2b, the freeze point of 3 M Zn(ClO_4_)_2_·6H_2_O aqueous electrolyte is as low as − 47 °C. After adding EG or hydrogel fabrication (HEA-3), the anti-freezing ability of the electrolyte was further improved significantly, with the frozen point both below − 60 °C. The anti-freeze abilities of electrolytes indicated that the coordination environment of H_2_O is dramatically interrupted in HEA-3 electrolyte compared to that in pure water [38].

Thermogravimetric analysis (TGA) was conducted to assess the thermal stability and water retention capacity of the HEA-3 electrolyte. As shown in Figs. 2c and S4, the weight loss of PAM/CMC/EG gels containing EG is relatively lower than that of PAM/CMC gel (38% vs. 46%) in the temperature range of 30–100 °C, suggesting that the strong hydrogen bond between EG and water molecules can decelerate the water evaporation [39]. Meanwhile, HEA-3 electrolyte has the lowest mass loss of 33% in the same temperature range. With the addition of Zn(ClO_4_)_2_ salts in PAM/CMC/EG system (HEA-3), the solvation effect of Zn^2+^ by surrounding water molecules further hinders the HBs formation among water, reducing the content of free water in the electrolyte system, and thus improving the thermal stability and water retention capacity of the electrolyte.

Figure 2d depicts the morphologies of pure PAM, PAM/CMC, PAM/CMC/EG, and HEA-4 electrolytes, respectively. The pure PAM hydrogel exhibits numerous irregular interconnected pore structures, which are conducive to ion conduction. The PAM/CMC hydrogel forms a semi-interpenetrating network polymer with irregular pore structures due to the physically interweaving and entanglement between CMC molecular chains and PAM molecular chains. However, with the introduction of EG, the pore structure transforms from disorder to order because of the intermolecular interaction between polymer chains and EG molecules. Moreover, the pore structure may collapse upon the introduction of the excessive Zn salts in the HEA-4 electrolyte (Fig. S5b), as the dissociation of Zn^2+^ and ClO_4_^−^ reduces the degree of cross-linking between molecular chains [35]. A homogeneous tunnel-structured polymeric matrix, such as that of HEA-3, ensures uniform ion flux and facilitates even metal deposition.

Figures 2e, f and S6 show the stress–strain curves and mechanical properties of the gel electrolytes, respectively. The tensile strength of PAM hydrogel is 71.6 kPa, with a fracture elongation of 463%. With the addition of CMC, a semi-interpenetrating network structure forms between PAM and CMC polymers via strong HB interactions between their molecular chains and physical entanglement of the chain segments. Consequently, the tensile strength and fracture elongation of the PAM/CMC gel increase to 178.2 kPa and 573%, respectively. Upon introducing EG, the tensile strength of the hydrogel electrolyte decreases to 143.3 kPa, while the fracture elongation increases to 1529%. This is because the multiple HB interactions among EG, PAM, and CMC molecular chains enhance the ductility of the hydrogel electrolyte. However, due to the dissociation effect of Zn^2+^ and ClO_4_^−^, the tensile strength and fracture elongation of the HEA-1 electrolyte decrease to 93.6 kPa and 1282%, respectively [40]. Nevertheless, the HEA-3 electrolyte still exhibits a tensile strength of 46.7 kPa and a fracture elongation of 979%, making it suitable for use in electrochemical energy storage devices.

To evaluate the ion transport behavior in hydrogel electrolytes, the electrochemical impedance spectroscopy (EIS) test was conducted at room temperature (Figs. 2g and S7), and the corresponding ionic conductivity values are calculated by Eq. 2. As the content of Zn salts increases in electrolyte, the more dissociated Zn^2+^ and ClO_4_^−^ in water contribute to the ion transport improvement, resulting in a higher ionic conductivity of the HEA-3 for 41.74 × 10^–3^ S cm^−1^, compared to HEA-1 for 22.24 × 10^–3^ S cm^−1^. However, the ionic conductivity decreases with the concentration of Zn salt and further increases (HEA-4). The variation of ionic conductivity with Zn salt may be explained according to the following relationship [41], $\sigma = \sum {n_{{\text{i}}} q_{{\text{i}}} \mu_{{\text{i}}} }$, where *n*_i_, *q*_i_, and *µ*_i_ are the density, charge, and mobility of free ions. Initial ionic conductivity improvement arises from increased density of free ions (*n*_i_) with Zn(ClO_4_)_2_ salt addition. However, the loading of excessive Zn(ClO_4_)_2_ salt induces the ion aggregation and ion-pair formation, reduces both density of free mobile ions (*n*_i_) and the mobility of free ions (*μ*_i_), thereby decreasing the ionic conductivity. Besides, the excessive Zn salt in electrolyte may lead to the collapse of the pore channel (HEA-4, as seen in Fig. S5b), thus further hindering the ion conduction in HEA-4 electrolyte.

The Zn^2+^ transfer numbers of the Zn(ClO_4_)_2_ (aq), Zn(ClO_4_)_2_ + EG and HEA-3 electrolytes were further evaluated to investigate the transport capability of Zn ions in different electrolytes [42]. Combined with the equivalent circuit model (Fig. S8a), the Zn^2^⁺ transference number (*t*_Zn_^2^_⁺_) can be calculated via Eqs. 3 and 4. As calculated, the Zn||Zn symmetric cell employing aqueous Zn(ClO_4_)_2_ (aq) demonstrates a low *t*_Zn_^2^⁺ of 0.47 (Fig. S8b). In contract, the EG-modified electrolyte (Zn(ClO_4_)_2_ + EG) achieves an enhanced *t*_Zn_^2^⁺ of 0.66 due to preferential Zn^2^⁺ coordination with EG over H₂O, which accelerates ligand exchange dynamics around Zn^2^⁺ ions, thereby enhancing the Zn^2+^ ions transport in the EG-containing electrolytes (Fig. S8c) [31]. Notably, the symmetrical Zn||Zn cell with HEA-3 electrolyte can provide a superior Zn^2+^ transference number of 0.89 (Zn^2+^ ionic conductivity: 37.15 × 10^–3^ S cm^−1^) (Fig. 2h). This high Zn^2+^ transfer number in HEA-3 electrolyte can be attributed to the rich interconnected pore channels within the electrolyte, providing numerous pathways for the migration of Zn ions, and the interactions between Zn^2+^ and abundance of carboxyl functional groups (COO–) on the polymeric matrix, inducing the effective transportation of Zn ions [43]. A high Zn^2+^ transfer number can enormously alleviate the concentration polarization and promote uniform deposition of zinc ions [44].

Considering the anti-freezing property of as-prepared electrolyte, the ionic conduction behavior at low temperature was investigated via EIS test and calculation (Figs. 2i and S9). It demonstrated that the HEA-3 electrolyte could maintain an ionic conductivity as high as 4.12 × 10^–3^ S cm^−1^ even at − 50 °C and connect a circuit successfully, enabling LED to emit blue light even in an ultra-low-temperature environment at − 60 °C (Fig. S10). Compared to the low-temperature ionic conductivity of start-of-the-art gel electrolytes (Table S1), our electrolyte shows a superior anti-freezing ability, ascribing to the successful coordination regulation of water molecule via delicate cross-linked tunnel-structured hydrophilic matrix for gel electrolyte.

### Reversibility of Zn Anodes in Cells with HEA-3 Electrolytes

**Fig. 3:**
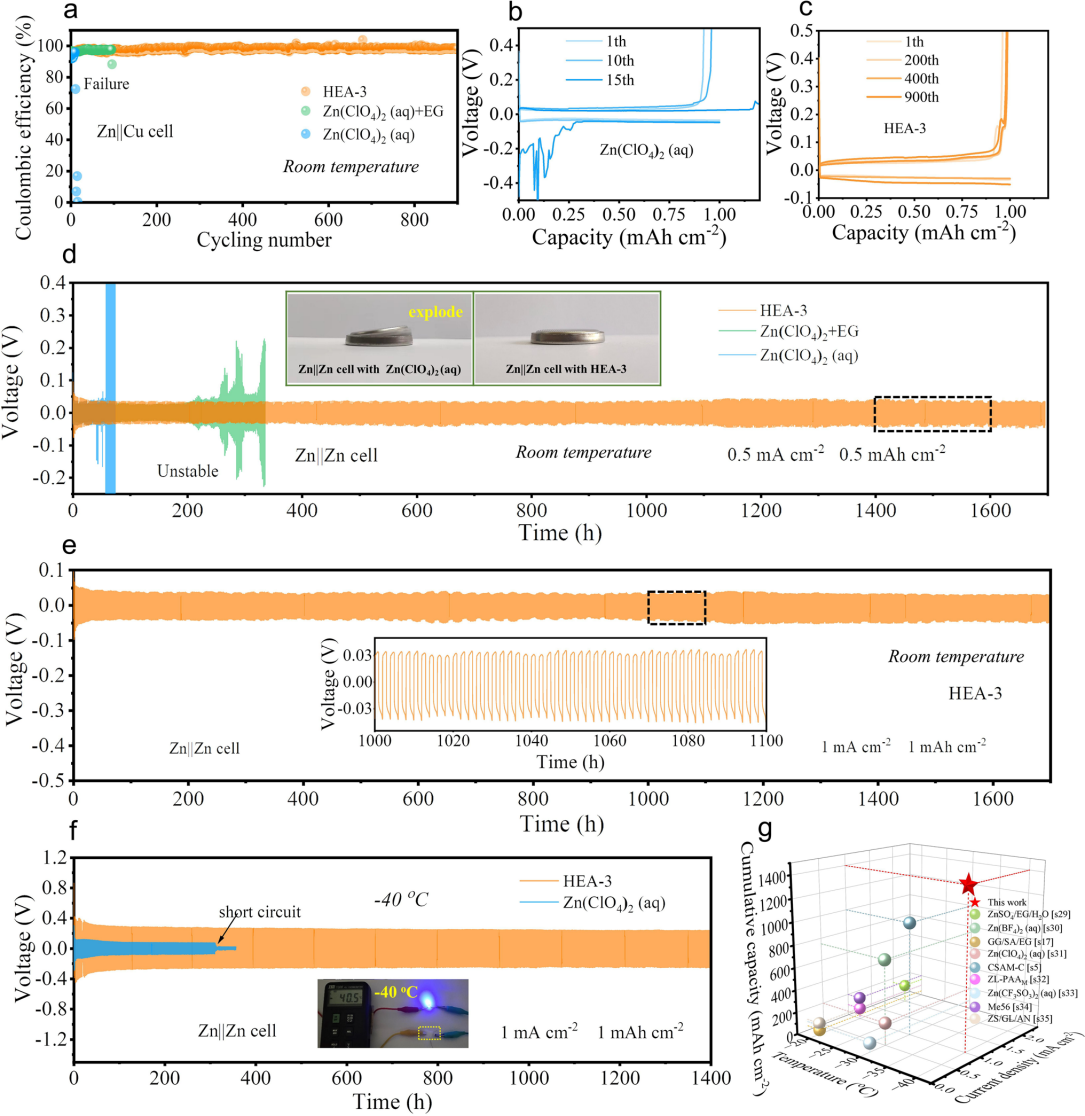
**a** Cycling performance of Zn||Cu cell at room temperature, and corresponding capacity–voltage curves in the **b** aqueous electrolyte, and **c** HEA-3 electrolyte at different cycles at a current density of 1 mA cm^−2^ with a fixed capacity of 1 mAh cm^−2^. **d** Room-temperature cycling performance of Zn||Zn cell with different electrolytes at a current density of 0.5 mA cm^−2^ with a fixed plating capacity of 0.5 mAh cm^−2^. Cycling performance of Zn|HEA-3|Zn cell (current density = 1 mA cm^−2^, fixed plating capacity = 1 mAh cm^−2^, DOD = 3.42%) at **e** room temperature and **f** − 40 °C. **g** Comparison of low-temperature performance of Zn||Zn cells with different reported electrolytes

The reversibility of Zn anodes in Zn||Cu cells with HEA-3 electrolyte was evaluated by the long-term galvanostatic charge–discharge (GCD) test. The initial Coulombic efficiency of the Zn||Cu cell with Zn(ClO_4_)_2_ (aq) as electrolyte is 91.7%, which is the lowest one compared to the cells using Zn(ClO_4_)_2_ (aq) + EG and HEA-3 electrolyte (Fig. 3a). Subsequently, the coulombic efficiency varies significantly, and the cells cannot sustain more than 15 charge/discharge cycles in aqueous electrolyte (Fig. 3b) due to the severe growth of Zn dendrites and hydrogen evolution reactions. Although the introduction of EG into Zn(ClO_4_)_2_ (aq) electrolyte improved the cycling stability from 15 to 97 cycles (Fig. S11), it could not fully suppress side reactions referring to HER and dendrite growth. In comparison, the HEA-3 electrolyte, when integrated with a PAM/CMC polymeric matrix, demonstrates outstanding cycling reversibility and stability, achieving a coulombic efficiency of up to 99.4% over 900 cycles. (Fig. 3a, c). The stripping-plating performance shown in Figs. 3d and S12 further confirms that the  Zn||Zn symmetric cells with the HEA-3 electrolyte can offer cycle stability for more than 1700 h under the current density of 0.5 mA cm^−2^ (0.5 mAh cm^−2^) at 25 °C. And this stability can be attributed to the reconstructed HB network in electrolyte, which restricts free water molecules and inhibits side reactions and dendrite growth during cycling process. On the contrary, the symmetrical cells using Zn(ClO_4_)_2_ (aq) and Zn(ClO_4_)_2_ (aq) + EG as electrolyte can only sustain 42 and 250 h of cycling, respectively, before short-circuiting or exploding due to the uncontrollable dendrite formation and side reactions. Even at a high current density (1 mA cm^−2^), the Zn||Zn cells employing HEA-3 as electrolyte still exhibit the remarkable electrode/electrolyte interfacial stability and suppressed HER, maintaining a stable polarization voltage over 1700 h (Fig. 3e). Moreover, the EIS plots of Zn||Zn cells with HEA-3 electrolytes after different cycles are shown in Fig. S13. The charge-transfer resistance of the cell increase initially during cycling and then will be stable after certain cycles. This phenomenon signifies the formation of a stable SEI layer formed at the electrolyte–Zn anode interface, which contributes to the excellent electrochemical stability of Zn plating/stripping in the HEA-3 electrolyte [4, 45, 46]. To further evaluate the zinc utilization, the Zn|HEA-3|Zn symmetric cell was subjected to charge–discharge testing at a high current density of 5 mA cm^−2^ with a fixed plating capacity of 5 mAh cm^−2^ (Fig. S14). According to Eq. 5, the depth of discharge (DOD) can be calculated as 17.1% [26]. Moreover, the room-temperature cycling stability of Zn|HEA-3|Zn symmetric cell also exhibits a good competitiveness compared to other Zn(ClO_4_)_2_-based electrolyte system (Table S2) [S23-S28]. Notably, as exhibited in Fig. 3f, even at − 40 °C, the Zn||Zn cell with HEA-3 electrolyte shows a low polarization voltage (250 mV) even at high current density of 1 mA cm^−2^ and can perform stable charge/discharge for more than 1400 h, demonstrating an outstanding low-temperature cycling performance, which are superior to state-of-the-art reported electrolytes at subzero temperatures (Fig. 3g, Table S3) [S5, S17, S29–S35]. Conversely, Zn||Zn cells with Zn(ClO_4_)_2_ aqueous electrolyte suffer short circuits after just 311 h of charging–discharging cycles. This can be attributed to severe concentration polarization at low temperatures, leading to uneven deposition of Zn^2+^ and promoting the growth of Zn dendrites.

To further evaluate the low-temperature reversibility of Zn stripping/plating behavior in HEA-3 electrolyte, Zn|HEA-3|Cu asymmetric cells were assembled and cycled at − 40 °C (Fig. S15a). The cell maintains a high Coulombic efficiency (CE) of 99.8% over 160 cycles. The corresponding galvanostatic charge/discharge (GCD) curves (Fig. S15b) present nearly identical voltage profiles across cycles, further confirming the robust electrochemical reversibility and uniform Zn deposition under the low-temperature conditions.

**Fig. 4:**
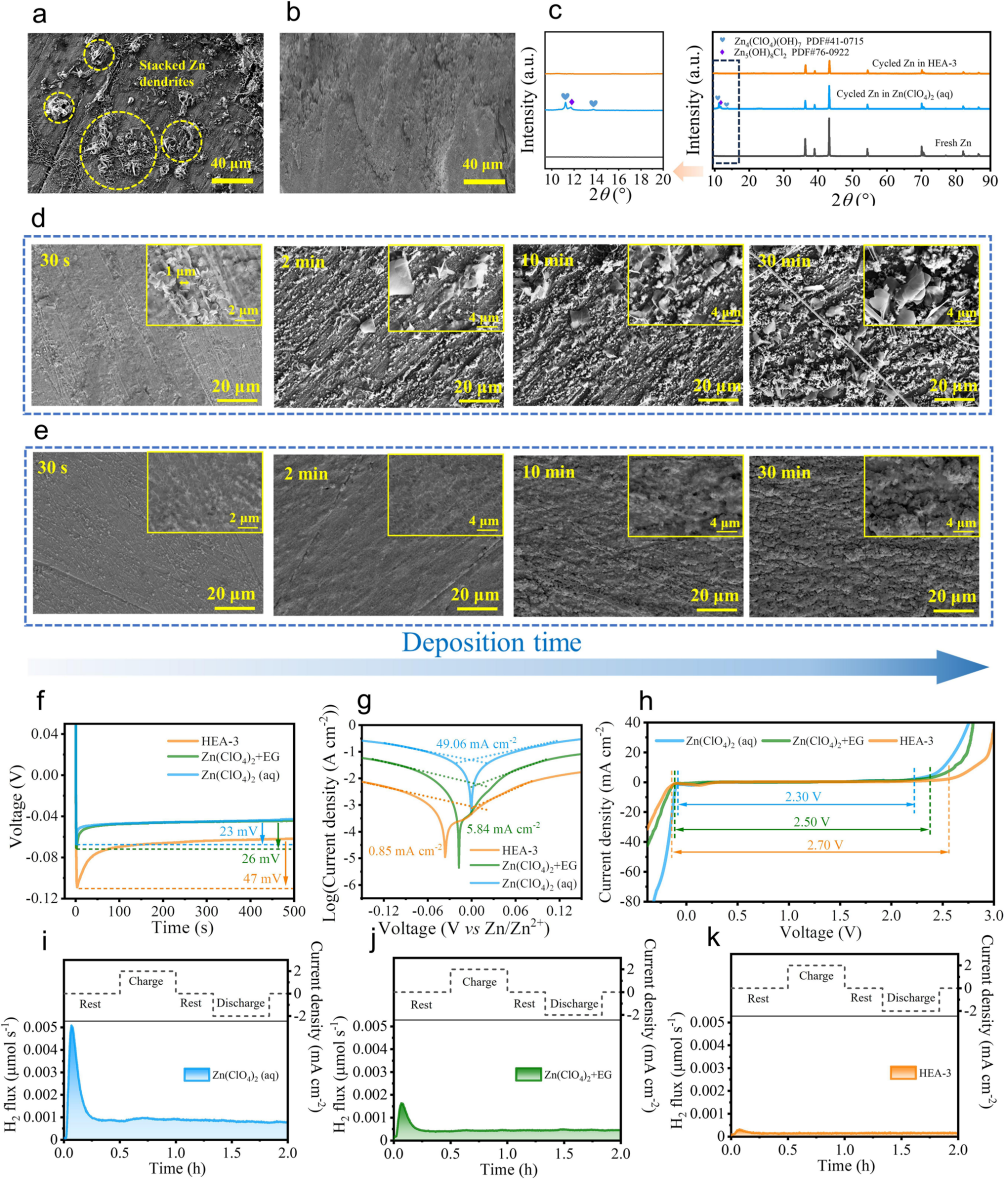
Surface morphology of cycled Zn anode in the **a** aqueous electrolyte (0.5 mAh cm^−2^), and **b** HEA-3 electrolyte (0.5 mAh cm^−2^). **c** XRD pattern of cycled Zn anodes in different electrolytes. The surface morphologies evolution of Zn deposits in the **d** aqueous electrolyte and **e** HEA-3 electrolyte. **f** Nucleation overpotential, **g** Tafel polarization curves, and **h** linear sweep voltammetry of cells in the Zn(ClO_4_)_2_ (aq), Zn(ClO_4_)_2_ + EG, and HEA-3 electrolyte. The in situ DEMS measurements of Zn||Zn symmetrical cells with **i** Zn(ClO_4_)_2_ (aq) electrolytes **j** Zn(ClO_4_)_2_ + EG electrolytes **k** HEA-3 electrolytes along with the corresponding current density–time curves

To further verify the function mechanism of the HEA-3 electrolyte on the cycling stability of Zn anodes, the surface morphologies and microstructures of cycled Zn anodes were investigated. The cycled Zn anode with Zn(ClO_4_)_2_ aqueous electrolyte exhibits a rough surface with irregularly stacked Zn dendrites (Figs. 4a and S16a), which can potentially lead to internal short circuits and accelerating cell failure. In contrast, the surface of cycled Zn with the HEA-3 as electrolyte is smooth and dense, as shown in Figs. 4b and S16b, indicating the effective dendrite inhibition. The XRD patterns of cycled Zn anodes in different electrolytes are illustrated in Fig. 4c.

Several obvious peaks located at 11.2° and 13.7° can be attributed to the common by-product Zn_4_ClO_4_(OH)_7_ (PDF#41-0715), while the peak around 11.7° can be ascribed to the Zn_5_(OH)_8_Cl_2_ (PDF#76-0922) by-product. These by-products are likely generated by electrochemical reaction processes, as reported in previous studies [47, 48].

11 $$5{\text{Zn}}^{2 + } + 2{\text{ClO}}_{4}^{ - } + 8{\text{H}}_{2} {\text{O}} + 16{\text{e}}^{ - } \to {\text{Zn}}_{5} ({\text{OH}})_{8} {\text{Cl}}_{2} + 8{\text{OH}}^{ - }$$

12 $$4{\text{Zn}} + {\text{ClO}}_{4}^{ - } + 8{\text{H}}_{2} {\text{O}} \to {\text{Zn}}_{4} {\text{ClO}}_{4} ({\text{OH}})_{7} + 4{\text{H}}_{2} \uparrow + {\text{OH}}^{ - }$$

13 $$4{\text{Zn}}^{2 + } + {\text{ClO}}_{4}^{ - } + 7{\text{OH}}^{ - } \to {\text{Zn}}_{4} {\text{ClO}}_{4} ({\text{OH}})_{7}$$

In the Zn(ClO_4_)_2_ aqueous electrolyte, owing to the HER and corrosion, a lot of OH^−^ can be generated at the electrolyte–electrode interface, leading to the formation of non-uniform insulating by-products, such as Zn_4_ClO_4_(OH)_7_ and Zn_5_(OH)_8_Cl_2_. These insulating by-products on the surface of Zn anode will deteriorate Zn^2+^ plating behavior, ultimately resulting in Zn dendrite growth. In contrast, there were no characteristic peaks relating to harmful by-products for Zn anodes with HEA-3 electrolyte, indicating that the HEA-3 electrolyte can promote the interfacial stability with effective suppression of HER and corrosion reactions, thereby enhancing the reversibility of Zn anodes over cycling. Besides, the surface of cycled Zn anodes at − 40 °C also exhibits a similar result: a uniform and dense surface without other by-products (Fig. S17a, b). In addition, XRD analysis of cycled Zn anodes reveals electrolyte-dependent crystallographic evolution during cycling. Fresh Zn exhibits a dominant (002) crystal plane (*I*_(002)_/*I*_(100)_ = 2.34), while cycling in aqueous Zn(ClO_4_)₂ electrolyte reduces this ratio to 2.00. Conversely, the HEA-3 electrolyte enhances (002) orientation (*I*_(002)_/*I*_(100)_ = 3.12) (Fig. S18a). This result indicated that the HEA-3 electrolyte can guide the Zn deposition along (002) crystal plane during the long-term cycling. Generally, the high-activation-energy (002) plane inhibits HER and corrosion side reactions during cycling. Moreover, the hexagonal (002) crystal plane with parallel alignment to the Zn surface facilitates homogeneous Zn^2+^ deposition, and effectively restrain Zn dendrites growth (Fig. S18b) [49, 50]. Therefore, the high *I*_(002)_/*I*_(100)_ value of cycled Zn in the HEA-3 electrolyte is contributing to obtain a smooth and compact surface without by-products.

To better reveal the deposition behavior of Zn in different electrolyte systems, the evolution of deposition morphologies of Zn during plating processes were monitored and recorded (deposition current density = 2 mA cm^−2^, shown in Fig. 4d, e). For the deposition of Zn in Zn(ClO_4_)_2_ aqueous electrolyte, the initial dendrites (a size of approximately 1 µm) are relatively uniform. However, as the deposition capacity increases, and the non-uniform dendrites form resulting in larger bulk lamellated stacked structure (Fig. 4d). In contrast, homogenous and fine Zn nanosheets with a size of about 0.4 µm form on the surface of Zn anode at the initial stage of deposition. As the deposition time increases, the morphologies of deposition turn into a fine swell cluster instead of large sheet stacked dendrites. This is attributed to the ordered and interconnected pore structure matrix in HEA-3 electrolyte, which can hinder the tips aggregation of Zn^2+^, and thus suppress the thick dendrites growth. Moreover, the in situ optical microscopy combined with a high plating current density of 5 mA cm^−2^ was performed to visually highlight the modulated Zn deposition behavior in different electrolytes. The results as shown in Fig. S19, distinct Zn protrusions formed on the Zn surface within 20 min in the Zn(ClO_4_)_2_ (aq) electrolyte, followed by rapid dendritic growth during electrochemical plating process. With the incorporation of EG additives, the growth rate of Zn dendrites was slightly restrained in the Zn(ClO_4_)_2_ + EG electrolyte, although localized irregularities still persist. In contrast, in the HEA-3 electrolyte system, the nucleation of Zn appeared smooth and compact, and no dendrites were observed throughout the whole plating process. These phenomena indicated that the HEA-3 electrolyte could enhance the stability of the Zn anode by constraining the water within a highly hydrophilic matrix network, restraining the side reactions, and regulating the Zn deposition. Consequently, the HEA-3 electrolyte ensures the long-term cycling stability of the cell. The growth thermodynamics of the Zn nucleus in the initial plating process are also investigated in Fig. 4f. Compared with the Zn in aqueous electrolyte (23 mV), Zn in HEA-3 electrolyte has a larger nucleation overpotential (47 mV). According to Eq. 6 [27, 28], the critical Zn nuclei radius (*r*) is inversely proportional to the nucleation overpotential (*η*), and this indicating that smaller nucleation sites for Zn plating in HEA-3 electrolyte leads to uniform deposition of Zn. This result is consistent with that of Zn plating morphologies in Fig. 4d, e. The formation of the homogeneous, smooth, and dense Zn deposition can effectively inhibit the unfavorable dendrite formation to the maximum extent [51].

To demonstrate the anti-corrosion ability of the Zn anode, the Tafel polarization curves in different electrolyte system shown in Fig. 4g. As seen, the corrosion current in the Zn(ClO_4_)_2_ + EG electrolyte (5.84 mA cm^−2^) is far lower than that in the Zn(ClO_4_)_2_ aqueous electrolyte (49.06 mA cm^−2^), indicating that the electrostatic interaction between EG and H_2_O can improve the coordination environment of H_2_O molecules, thus suppressing the electrochemical activity of H_2_O molecules. For the Zn||Zn cell with the HEA-3 electrolyte, the corrosion current further reduces to 0.85 mA cm^−2^, nearly seven times lower than that with Zn(ClO_4_)_2_ + EG electrolyte. And this indicates that the coordination environment of H_2_O molecules is further regulated by strong HBs network of hydrogel matrix. Consequently, the activity of water molecules and the H^+^ diffusion behavior are further restrained in HEA-3 electrolyte, thus reducing the corrosion of Zn and improving the electrolyte compatibility with the Zn anode [52]. The electrochemical stability window of the developed hydrogel was further evaluated with LSV to confirm the coordination regulation effect of water by hydrogel framework. As shown in Fig. 4h, the overall stability window of the cell with HEA-3 is significantly expanded to 2.7 V, which is much wider than those of cells in Zn(ClO_4_)_2_ + EG and Zn(ClO_4_)_2_ electrolyte (2.5 and 2.3 V, respectively). This improvement is mainly attributed to the enhanced hydrogen bond between water and hydrogel matrix, which dramatically decreases the content of free water and suppresses H^+^ diffusion behavior, thereby effectively restraining both the HER and OER.

The HER behaviors of Zn metal anodes in different electrolytes were systematically investigated using in situ differential electrochemical mass spectrometry (DEMS) [53]. Employing this technique, H₂ generation during Zn plating/stripping was quantified in Zn||Zn symmetric cells cycled at 2 mA cm⁻^2^/1 mAh cm⁻^2^ per half-cycle. The upper panel of Fig. 4i–k (upper) displays the current density-time profiles of the Zn||Zn symmetrical cells, while Fig. 4i–k (below) reveals the time-dependent H₂ evolution rates in different electrolytes. The Zn||Zn symmetrical cells with different electrolyte were sealed for 30 min to evaluate its hydrogen production after being statically rested. As shown in Fig. 4i, in the aqueous Zn(ClO_4_)_2_ electrolyte, H₂ signals emerge after 30 min of resting and escalate dramatically, consistency with severe HER, with a relatively high H_2_ flux (0.93 × 10^–3^ μmol s^−1^) during the charge–discharge process. While, the reduced H_2_ evolution for EG-modified electrolyte (Zn(ClO_4_)_2_ + EG) compared to the aqueous Zn(ClO_4_)_2_ electrolyte could attribute to EG-H₂O interactions that partially suppress the HER (Fig. 4j). Remarkably, there is no significant hydrogen evolution for the anodes in the HEA-3 electrolyte system in the seal resting period (half an hour), and a negligible H_2_ flux (0.11 × 10^–3^ μmol s^−1^) was detected throughout charge–discharge process (Fig. 4k). This phenomenon further demonstrates the exceptional HER suppression capability of HEA-3 electrolyte.

### Performance Evaluation of Zn-Based Devices with the HEA-3 Electrolytes

**Fig. 5:**
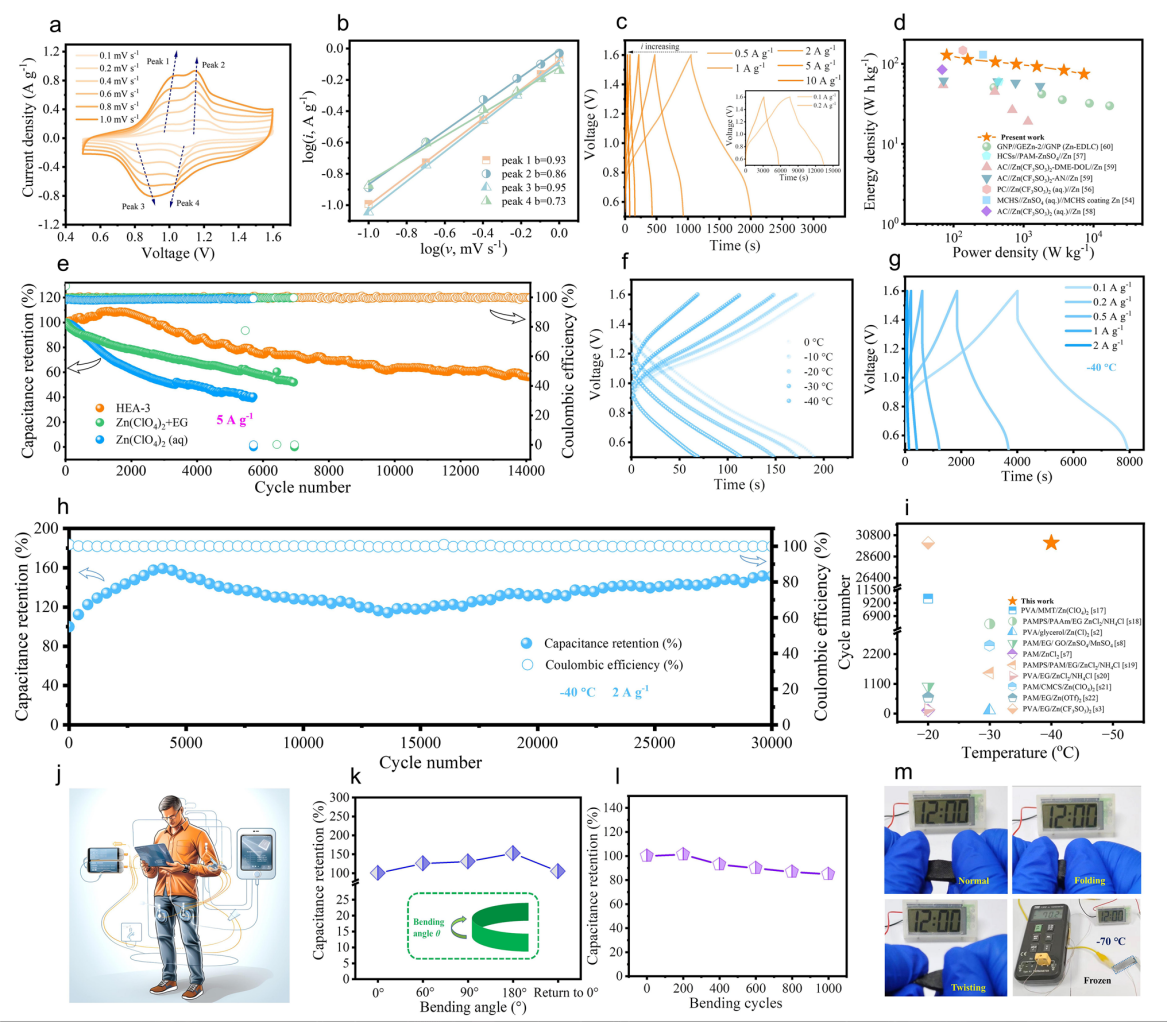
**a** CV curves of Zn||PANI devices under different scan rates. **b** The corresponding log (peak current) versus log (scan rate) plots at each redox peak. **c** GCD curves of Zn||PANI devices under different current densities, **d** Ragone plots of Zn-based energy storage devices. **e** Cycling performance of Zn||PANI devices with different electrolytes at a current density of 5 A g^−1^. **f** GCD curves of Zn||PANI devices at different temperatures under a current density of 2 A g^−1^. **g** GCD curves of Zn||PANI devices with different current densities at − 40 °C. **h** Cycling performance of Zn||PANI devices with a current density of 2 A g^−1^ at − 40 °C. **i** Comparison of low-temperature performance between the Zn||PANI device and others. **j** Application scenario of the flexible wearable energy storage devices. **k** Capacitance retentions of flexible Zn||PANI device under different bending conditions relative to the value without bending. **l** Capacitance retentions for various bending times at a bending angle of 180°. **m** Demonstration of the flexible Zn||PANI device can operate under various special conditions (normal, folding, twisting, and frozen at − 70 °C)

The Zn-based devices (Zn||PANI) were fabricated in order to evaluate the electrochemical behavior of the full cell. Figure 5a shows the CV curves of Zn||PANI devices with different scan rates. According to Dunn’s report, the electrochemical kinetics and charge/discharge mechanism can be revealed by current responses in CV curves at different scan rates [54]. The relationship between peak current (*i*) and scan rate (*v*) can be disclosed in Eqs.7 and 8 [54, 55]. It is recognized that the electrochemical reaction is a diffusion-controlled process if *b* = 0.5, whereas pseudocapacitance dominates the charge/discharge process if the parameter *b* = 1. Since the *b* values are obtained by the slope of the log(*v*)–log(*i*) plots, the calculated *b* values of peaks 1, 2, 3, and 4 are 0.93, 0.86, 0.95, and 0.73, respectively (Fig. 5b), indicating that the electrochemical kinetic for the Zn||PANI device is controlled by both diffusion and capacitive process and the surface capacitive contribution is the dominant one (Fig. S20a). With the increase of scan rate, the capacitive contribution can increase to 84% at 1.0 mV s^−1^ from 63% at 0.1 mV s^−1^ (Fig. S20b, Eqs. 9 and 10), revealing that this Zn||PANI device have favorable charge-transfer kinetics.

Figure 5c depicts the galvanostatic charge/discharge (GCD) curves of the Zn||PANI devices under various current densities (0.1 to 10 A g^−1^). It delivers a good rate capability of 363.5, 322.7, 303.8, 288.2, 275.7, 261.3, and 250.2 F g^−1^ at 0.1, 0.2, 0.5, 1, 2, 5, and 10 A g^−1^, respectively. In addition, the device exhibits a high power density of 7.315 kW kg^−1^ at 74.4 Wh kg^−1^ and a high energy density of 128.5 Wh kg^−1^ at 79.8 W kg^−1^, which surpass the previously reported Zn-based devices (Fig. 5d) [54, 56–60]. Figure 5e shows the cycling performance of the device with different electrolytes under a current density of 5 A g^−1^. As seen, the Zn||PANI devices with HEA-3 electrolyte show an outstanding cycling stability. When compared to the devices with Zn(ClO_4_)_2_ (aq) and Zn(ClO_4_)_2_ + EG as electrolyte, its capacity can retain at 70% of the initial capacity after long-term cycling over 8000 cycles and the coulombic efficiency keep nearly at 100% throughout the cycling process. Moreover, the cycle life can extend up to 14,120 cycles. Furthermore, the cycling performance of Zn||PANI devices with different electrolytes was evaluated at very low current density of 0.1 A g⁻^1^ to further assess reversibility and stability. Devices using Zn(ClO₄)₂ or Zn(ClO₄)₂ + EG electrolytes failed to charge normally at 0.1 A g⁻^1^ due to severe HER and corrosion (Fig. S21a). In contrast, the HEA-3-based device exhibited superior stability, retaining 58% capacity after 60 cycles at 0.1 A g⁻^1^ (Fig. S21b). The enhanced cycling stability and reversibility of the Zn||PANI devices with HEA-3 electrolyte can be attributed to the powerful inhibition of corrosion, HER, and dendrites by regulating the coordination environment of H_2_O molecules.

The low-temperature performance of the Zn||PANI device assembled with the HEA-3 electrolyte was evaluated by GCD technique. Figure 5f shows the GCD curves of the device with various temperatures at the current density of 2 A g^−1^. As the operation temperature decreases (− 20, − 30, and − 40 °C), the capacity is 235.8, 185.6, and 122.4 F g^−1^, respectively, and this phenomenon is mainly ascribed to the reduced ion conductivity and sluggish electrochemical kinetics at low temperatures. Nevertheless, the device could still deliver a relatively high specific capacity (122.4 F g^−1^ at 2 A g^−1^) even at − 40 °C. Figure 5g presents the rate capability of the Zn||PANI device with HEA-3 as electrolyte at temperature of − 40 °C. Owing to the outstanding anti-freezing and high low-temperature ion conductivity of HEA-3 electrolyte (as proved in Fig. 2i), the capacity of the device can be retained at 70% even at − 40 °C compared to the capacity value at room temperature. The long-term cycling stability of the device operated at low-temperature with HEA-3 as electrolyte are shown in Fig. 5h as well. It displays that the Zn-based device exhibits a good long-term cycling performance, maintaining reversible charge/discharge for more than 30,000 cycles at a large current density of 2 A g^−1^. The phenomenon of capacity increases at an early stage and can be ascribed to the self-activation process, in which the polarized ions diffuse at the electrolyte–electrode interface, and the interfacial compatibility can be improved during this process [56, 61, 62].

Remarkably, the Zn||PANI with HEA-3 electrolyte device demonstrates exceptional ultra-low-temperature operability, maintaining functionality at − 70 °C with GCD profiles under different current densities shown in Fig. S22a. At 0.05 A g⁻^1^, it delivers 192.7 F g⁻^1^ with a high Coulombic efficiency of 99.9%, while achieving 96.9 F g⁻^1^ with 100% Coulombic efficiency at 0.1 A g⁻^1^. In addition, the device retains 82% of its initial capacity after 400 cycles at 0.1 A g⁻^1^ under these extreme conditions (Fig. S22b). The low-temperature cycling performance of the device is superior to other Zn-based devices reported recently (Fig. 5i and Table S1) [S2, S3, S7, S8, S17-S22], and this outstanding cycling stability and excellent electrochemical performance at low-temperature pave a way for potential application in extreme environment.

Flexible wearable energy storage devices can provide more convenience to human life, as shown in Fig. 5j. To further expand the application scenario of the present idea, the flexible Zn||PANI device was assembled with the flexible PANI as cathode, HEA-3 hydrogel as electrolyte, and flexible Zn as anode as illustrated in Fig. S23. The capacitance retentions based on the charge/discharge test reveal an increase in capacity for the device with the bending angles from 0° to 180°. A 104.6% capacity retention of the initial value is achieved as bending angle returns to 0° (Figs. 5k and S24a), demonstrating its good mechanical flexibility and ionic conductive capability. The capacity fluctuation against the bending angle can be interpreted as below (Fig. 5k). By applying the external force during bending, the HEA-3 electrolyte undergoes deformation and penetrates into the flexible PANI cathode, enhancing the interfacial contact between the electrodes and electrolyte, and thus improving the capacity of the device. Figures 5l and S24b show the capacitance evolution of the flexible device along with different bending times at a bending angle of 180°. The capacitance retains 85.0% of its initial capacity even after bending 1000 times, demonstrating the excellent mechanical flexibility of the device. In addition, the flexible device can power an electronic watch under deformation conditions (folding and twisting) and even at an ultra-low temperature of − 70 °C, as evident in Fig. 5m and Video S1. These results further demonstrate the feasibility of AZMBs with high environmental adaptability (HEA-3) electrolyte as flexible energy storage devices for extreme temperature environments in the future.

## Conclusions

A facile and effective strategy for regulating the coordination environment regulation of water molecules is developed for achieve a highly environment-adaptable and high-stability flexible Zn devices. By fabricating a highly cross-linked tunnel-structured hydrophilic hydrogel polymer electrolyte, the continuous H bond between H_2_O molecules has been disrupted, and the H_2_O molecules are bounded in the reconstructed multiple H bond network created by the EG molecules, CMC, and PAM molecules chains. Consequently, the activity of H_2_O molecules and the transportation behavior of H^+^ have been restricted, suppression of HER, corrosion, and dendrites growth and enabling a high reversibility (CE = 99.4% in Zn||Cu cell) of Zn anode with the HEA-3 electrolyte. Meanwhile, the assembled flexible Zn||PANI device with the HEA-3 electrolyte also demonstrates high capacitance and ultralong cycling stability operating at both room temperature and ultra-low temperature (-70 °C) due to the regulation of coordination environment of water. Such results represent a significant advance toward promoting the application of prepared hydrogel electrolyte in flexible wearable devices and energy storage device for extreme environments.

## Supplementary Information

Below is the link to the electronic supplementary material.

Supplementary file1 (DOCX 3722 KB)

Supplementary file2 (MP4 9158 KB)
