# Supplementary material for: Regulating the Coordination Environment of H2O in Hydrogel Electrolyte for a High-Environment-Adaptable and High-Stability Flexible Zn Devices
Source: Nanomicro Lett. 2025 Jun 12;17:292. doi: 10.1007/s40820-025-01810-4 (PMC12162457; doi:10.1007/s40820-025-01810-4)
Supplement: Supplementary file 1 — Supplementary file1 (DOCX 3722 KB) [file 40820_2025_1810_MOESM1_ESM.docx]

Supporting Information for

**Regulating the Coordination Environment of H_2_O in Hydrogel Electrolyte for a High Environment-Adaptable and High-Stability Flexible Zn Devices**

Jianghe Liu^1,2^, Qianxi Dang^2^, Jodie Yuwono^3^, Shilin Zhang^3^, Zhixin Tai^1,^*, Zaiping Guo^3^, Yajie Liu^1,^*

^1^ Advanced Energy Storage Materials and Technology Research Center, Guangdong-Hong Kong Joint Laboratory for Carbon Neutrality, Jiangmen Laboratory of Carbon Science and Technology, Jiangmen, Guangdong 529199, P. R. China

^2^ Shenzhen Key Laboratory of Advanced Materials, School of Materials Science and Engineering, Harbin Institute of Technology, Shenzhen, Guangdong 518055, P. R. China

^3^ School of Chemical Engineering, Faculty of Sciences, Engineering and Technology, University of Adelaide, Adelaide, SA, 5005 Australia.

*Corresponding authors. E-mail: [liuyajie@hkustgz-jcl.ac.cn](mailto:liuyajie@hkustgz-jcl.ac.cn) (Yajie Liu); [taizhixin@hkustgz-jcl.ac.cn](mailto:taizhixin@hkustgz-jcl.ac.cn) (Zhixin Tai)

**Supplementary Figures and Tables**

**
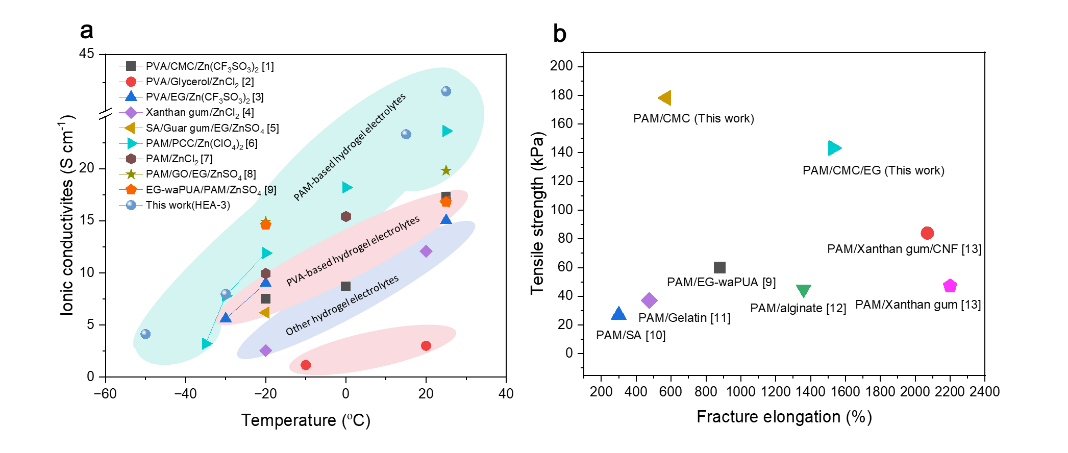
**

**Fig. S1 a** Wide-temperature ionic conductivities of various reported Zn-ion hydrogel electrolytes [S1-S9], **b** Mechanical properties of PAM-based hydrogel [S9-S13]


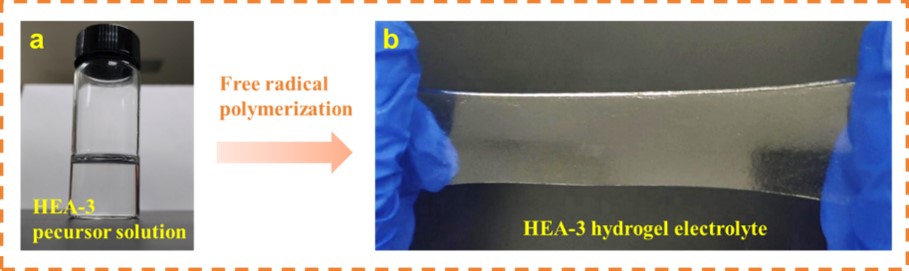


**Fig. S2** Optical photograph of (**a**) HEA-3 pecursor solution; (**b**) HEA-3 hydrogel electrolyte membrane


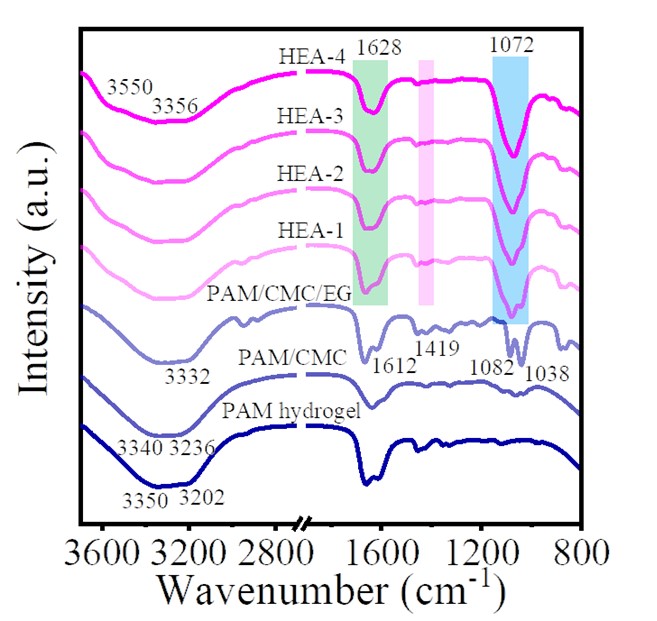


**Fig. S3** FTIR spectrum of the PAM, PAM/CMC, PAM/CMC/EG, and high environmentally adaptable hydrogel electrolytes (HEA-1, HEA-2, HEA-3, HEA-4)


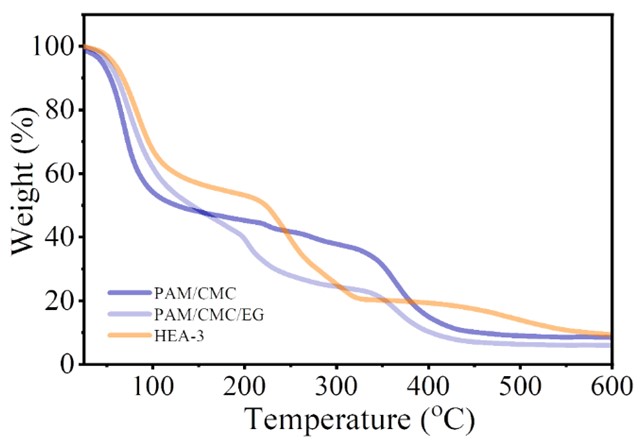


**Fig. S4** TGA curves of PAM/CMC, PAM/CMC/EG, and HEA-3 at a test range form 25 to 600 ºC

For the HEA-3 gel electrolyte, the weight loss in the temperature range of 100-220 ^ο^C can be attributed to the volatilization of ethylene glycol. The mass loss in the range 220-330 ^ο^C results from the decomposition of Zn(ClO_4_)_2_ salt and the carbonization of CMC, while the mass loss in the temperature range 400-570 ^ο^C is associated with the decomposition of the PAM.


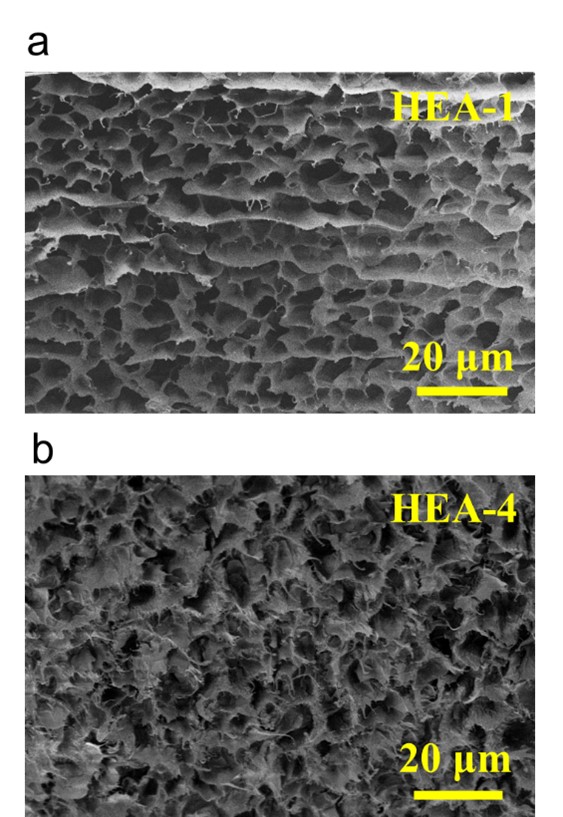


**Fig. S5** SEM images of **a** HEA-1 and **b** HEA-4 hydrogel electrolytes


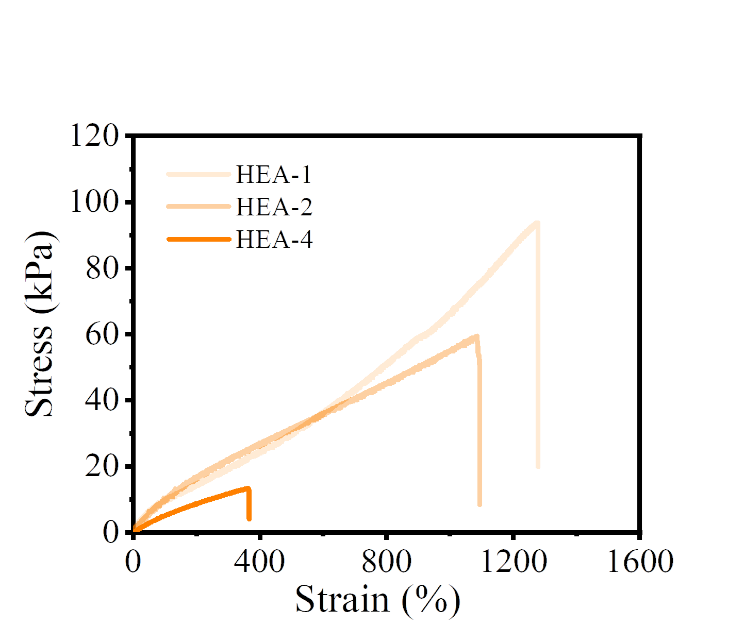


**Fig. S6** Strain-stress curves of the HEA-1, HEA-2 and HEA-4 hydrogel electrolytes

**
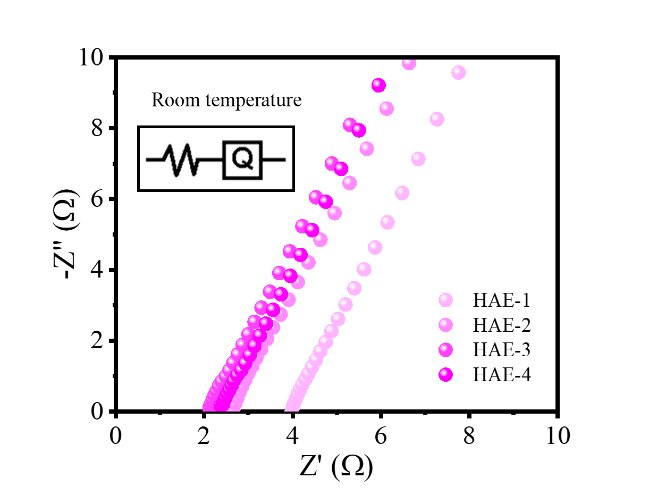
**

**Fig. S7** EIS plots of HEA hydrogel electrolytes measured at room temperature, with the corresponding equivalent circuit shown in the inset

The EIS plots of hydrogel electrolytes consistently feature a high-frequency real-axis intercept corresponding to bulk ionic resistance (R_b_), followed by a linear low-frequency response reflecting distributed capacitive behavior at electrode-electrolyte interfaces. The equivalent circuit (inset) incorporates R_b_ in series with a constant phase element (Q), which models non-ideal double-layer dynamics and interfacial heterogeneity [S14, S15].


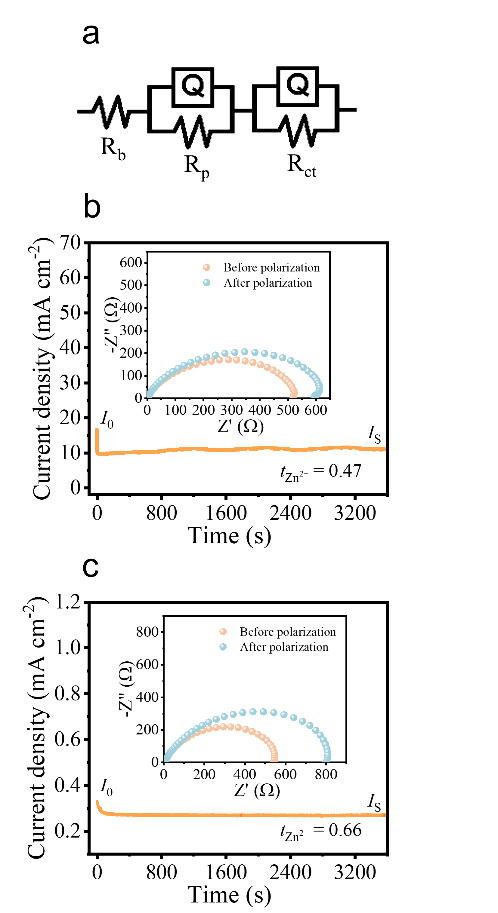


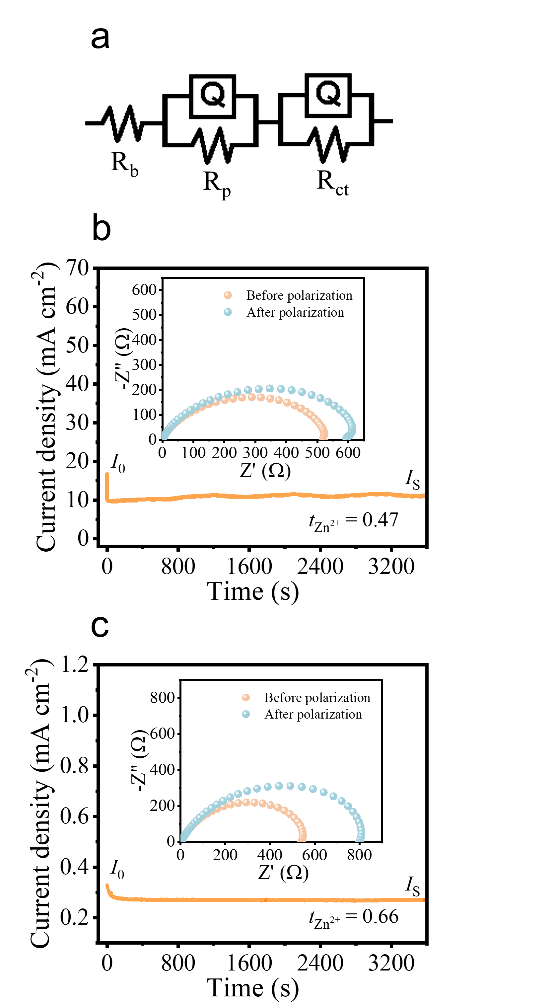

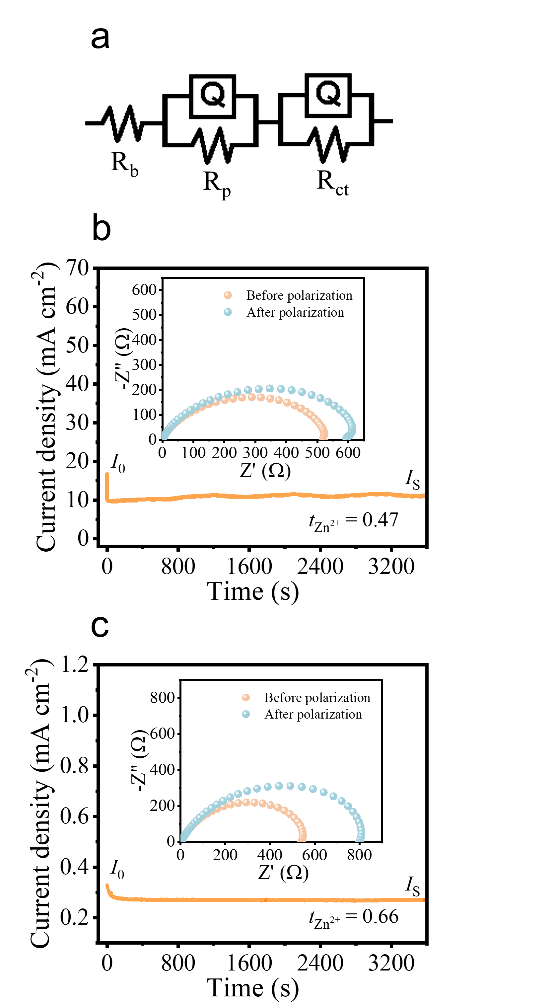


**Fig. S8** **a** the equivalent circuit model of Zn||Zn symmetric cell, and DC polarization curve of the cells with **b** Zn(ClO_4_)_2_ (aq) and **c** Zn(ClO_4_)_2_+EG electrolyte at room temperature (the inset shows the EIS plots of the corresponding Zn||Zn symmetric cell before and after polarization).

In the equivalent circuit, in the high-frequency region, the value of the first intersection between the impedance spectrum and the Z-axis represents the bulk resistance (R_b_) of the electrolyte. The first semicircle can be expressed by a parallel combination of the charge transfer resistor (R_ct_) and a constant phase element (Q). The second semicircle can be represented by a parallel combination of the passivating layer resistor (R_p_) and another constant phase element (Q) [S16]. **
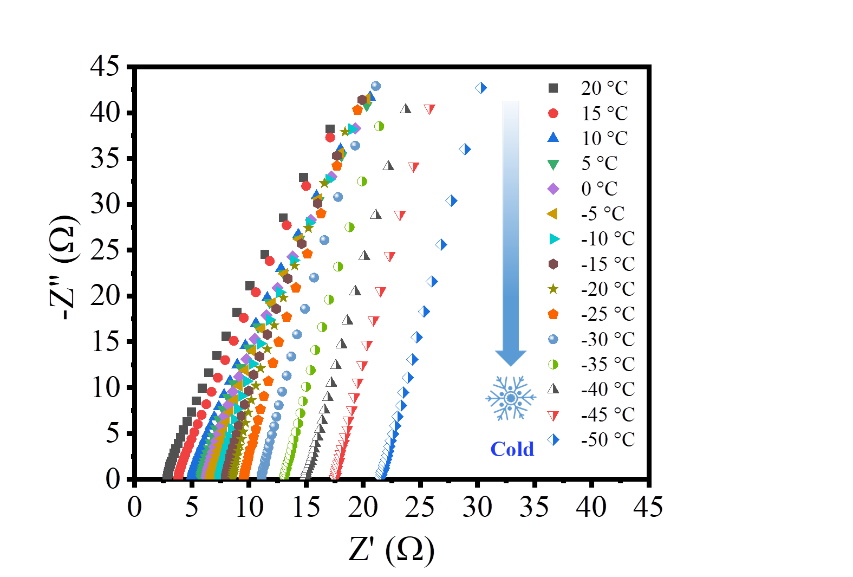
**

**Fig. S9** EIS plots of the HEA-3 hydrogel electrolyte at different temperatures


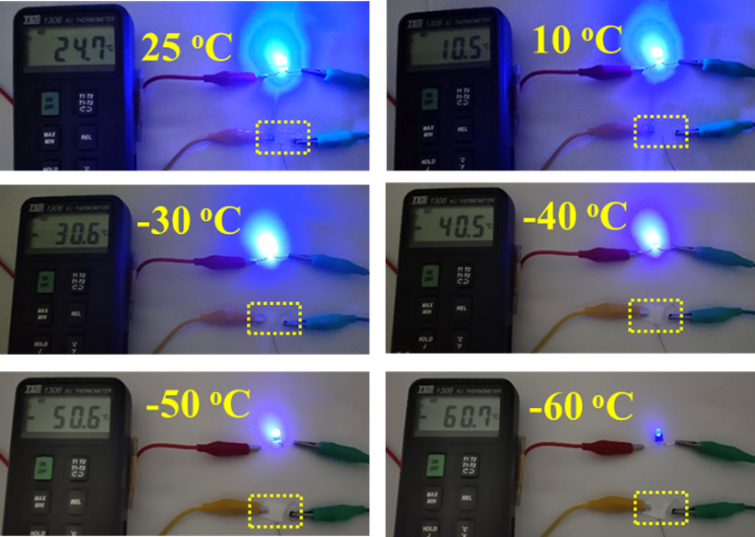


**Fig. S10** Low-temperature ionic conduction ability of the HEA-3 hydrogel electrolyte

**
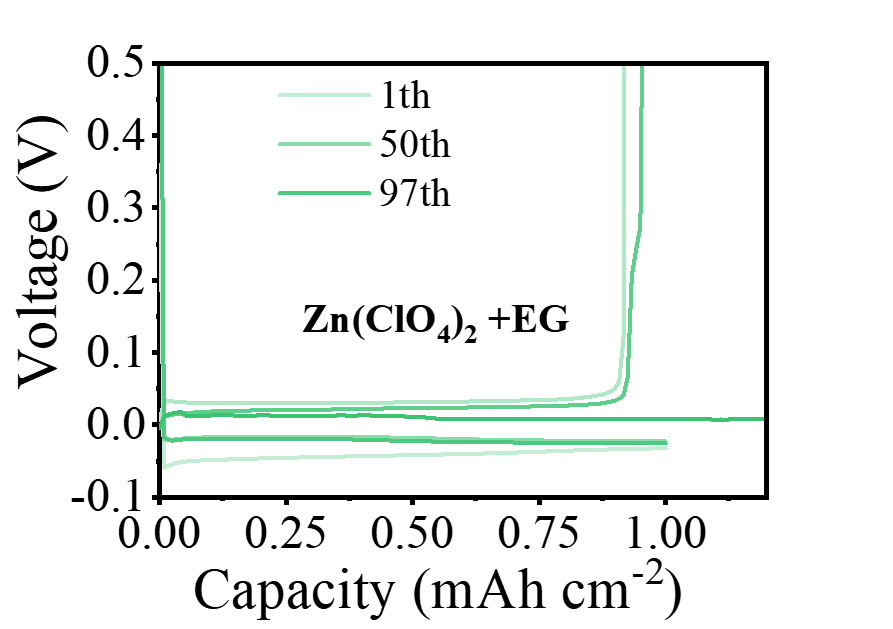
**

**Fig. S11** Capacity-voltage curves in the Zn(ClO_4_)_2_+EG electrolyte at different cycles at a current density of 1 mA cm^-2^ with a fixed capacity of 1 mAh cm^-2^


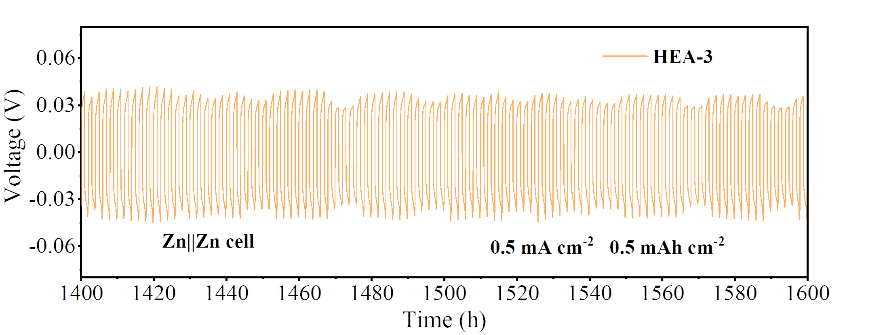


**Fig. S12** Magnification of the GCD curve of Zn||Zn cell with HEA-3 electrolytes at a current density of 0.5 mA cm^-2^ with a fixed plating capacity of 0.5 mAh cm^-2^
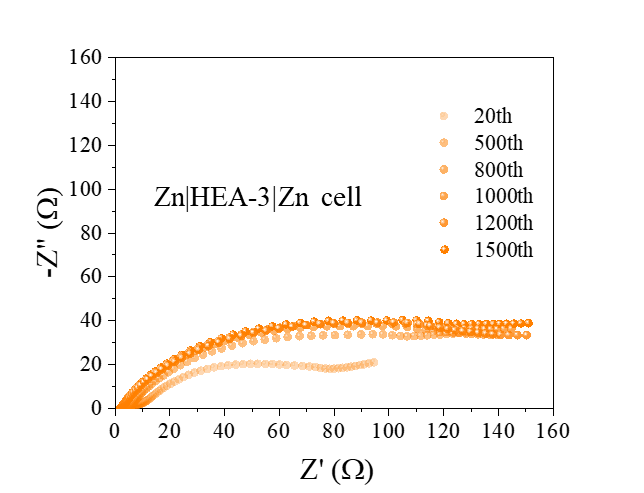


**Fig. S13** EIS plots of Zn|HEA-3|Zn symmetric cell after different cycles under GCD cycling (a current density of 1 mA cm^−2^ with a fixed capacity of 1 mAh cm^−2^) .


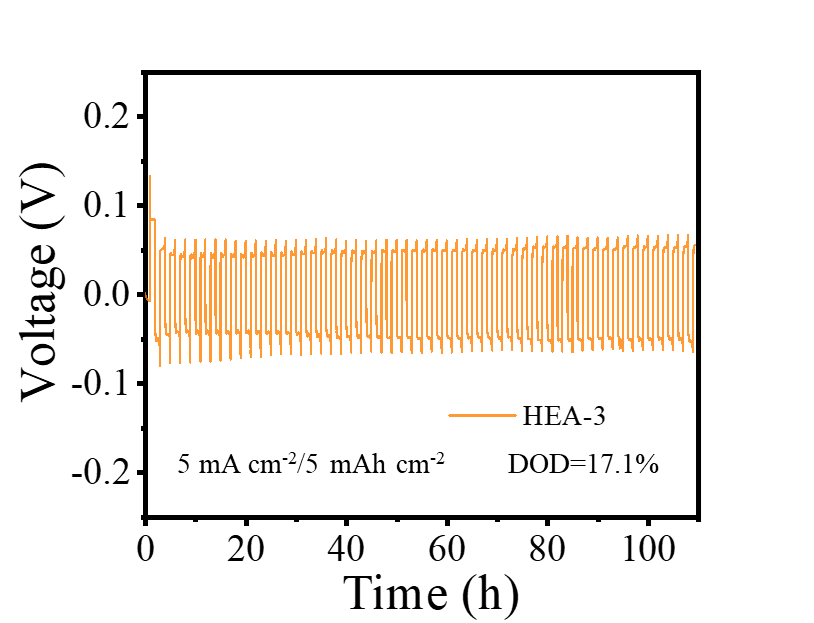


**Fig. S14** Room temperature cycling performance of Zn|HEA-3|Zn cell w at a current density of 5 mA cm^-2^ with a fixed plating capacity of 5 mAh cm^-2^


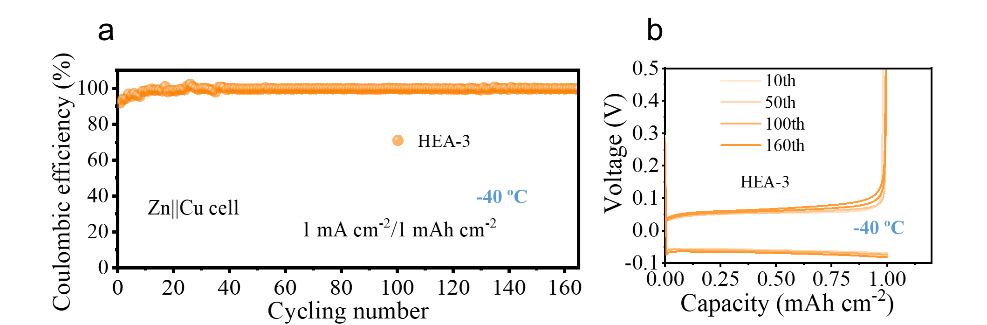


**Fig. S15** **a** Cycling performance of Zn|HEA-3|Cu cell at -40 ºC, and **b** corresponding capacity-voltage curves at different cycles at a current density of 1 mA cm^-2^ with a fixed capacity of 1 mAh cm^-2^

**
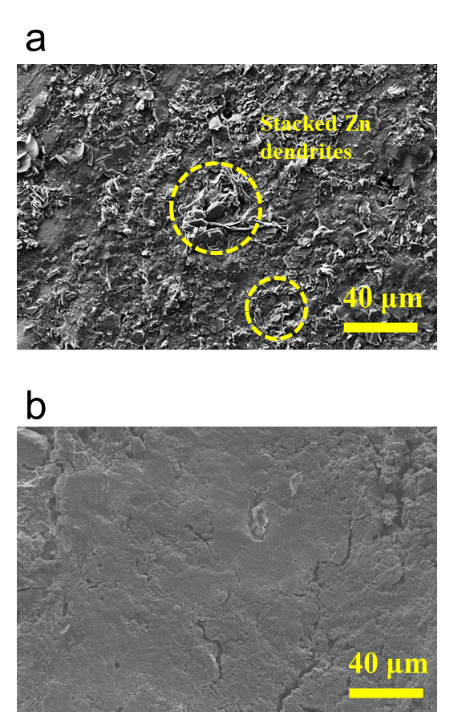

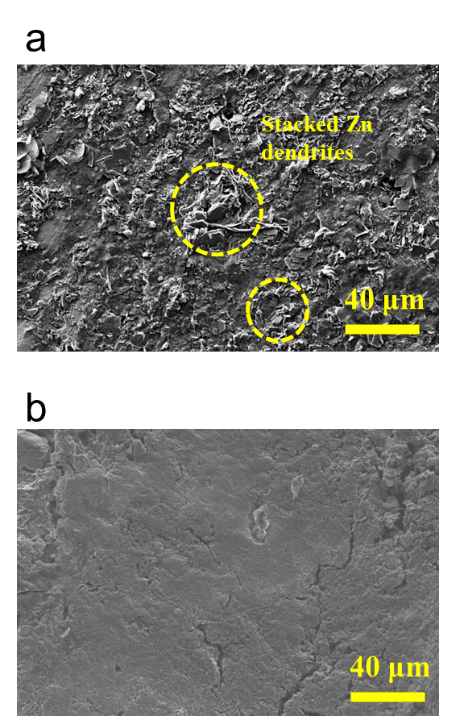
**

**Fig. S16** SEM images of cycled Zn anode of Zn||Cu cell in **a** aqueous electrolyte and **b** HEA-3 hydrogel electrolyte, under a current density of 1 mA cm^-2^ at room temperature

**
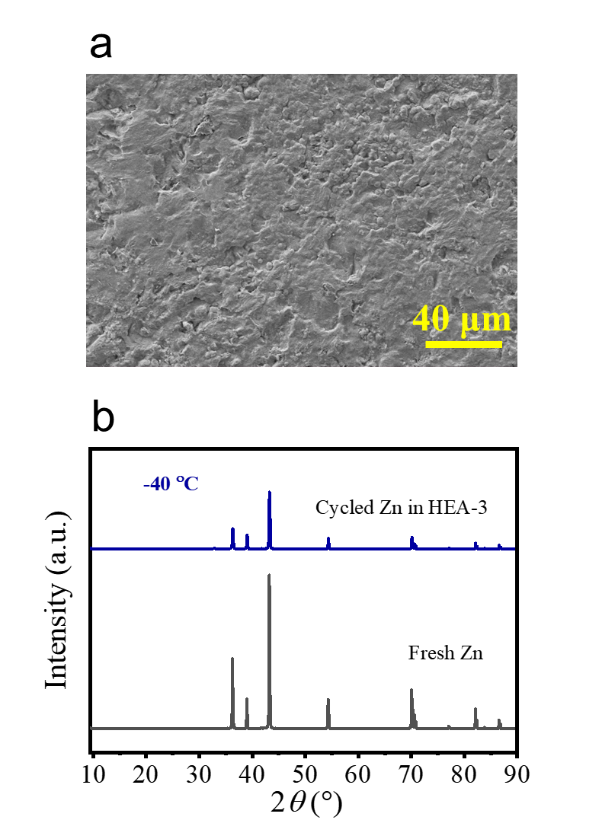

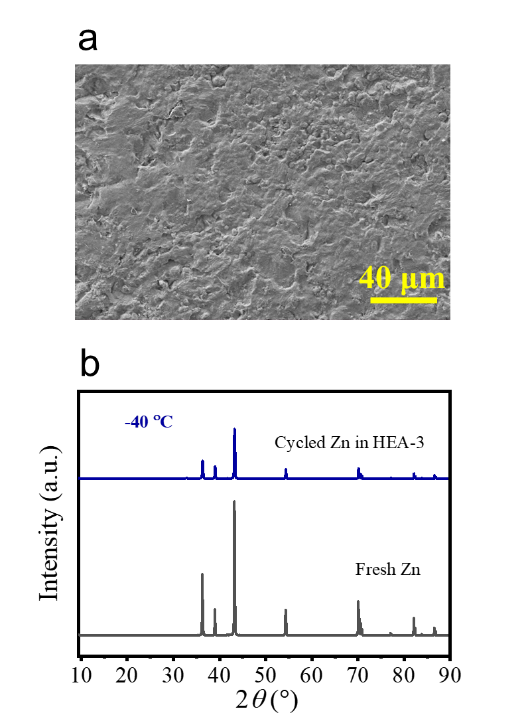
**

**Fig. S17** **a** SEM images and **b** XRD pattern of cycled Zn anode surface in HEA-3 electrolyte at -40 ºC


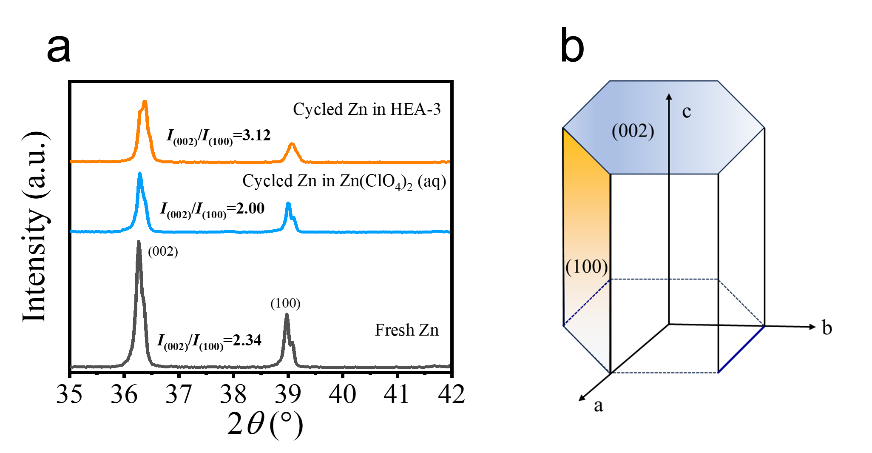


**Fig. S18** **a** XRD pattern of cycled Zn anodes in different electrolytes at room temperature. **b** Illustration of hexagonal structure of Zn

**
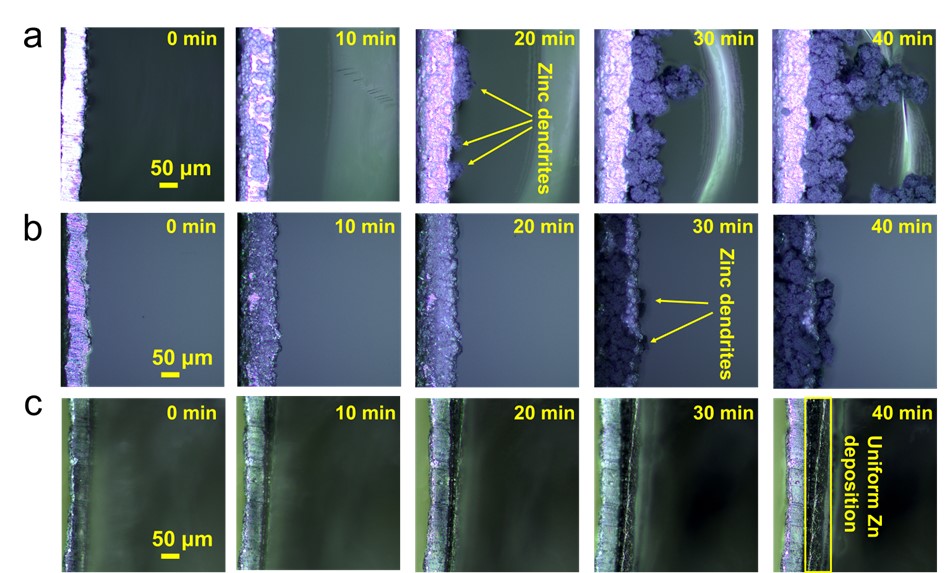
**

**Fig. S19** In-situ observation of Zn plating in Zn||Zn symmetric cell with **a** Zn(ClO_4_)_2_ (aq) electrolyte, **b** Zn(ClO_4_)_2_+EG electrolytes, **c** HEA-3 electrolyte (current density: 5 mA cm^-2^; scale bars: 50 µm)

**
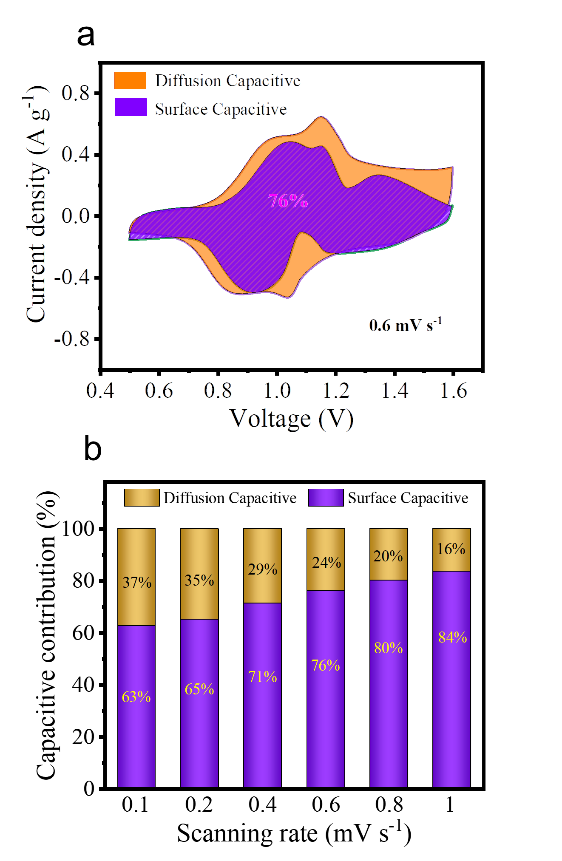

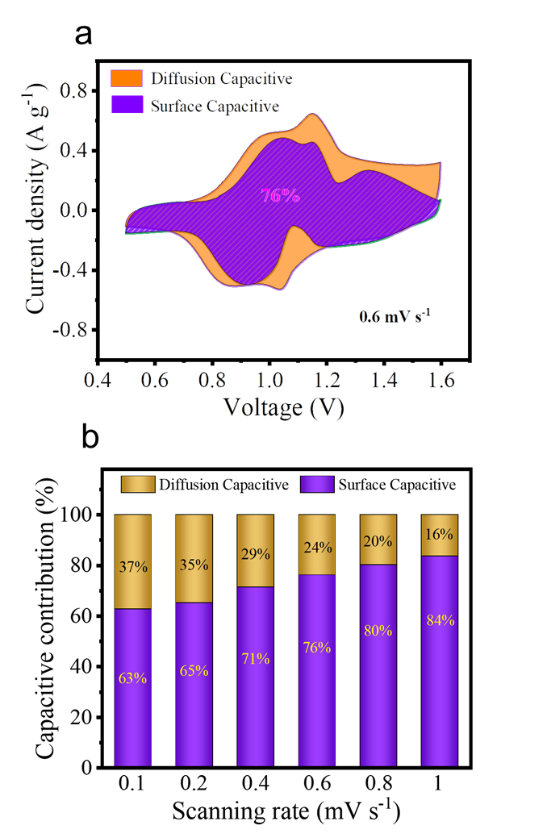
**

**Fig. S20** **a** Diffusion contribution and capacitive contribution at 0.6 mV s^-1^. **b** Normalized capacity contribution at different scan rates


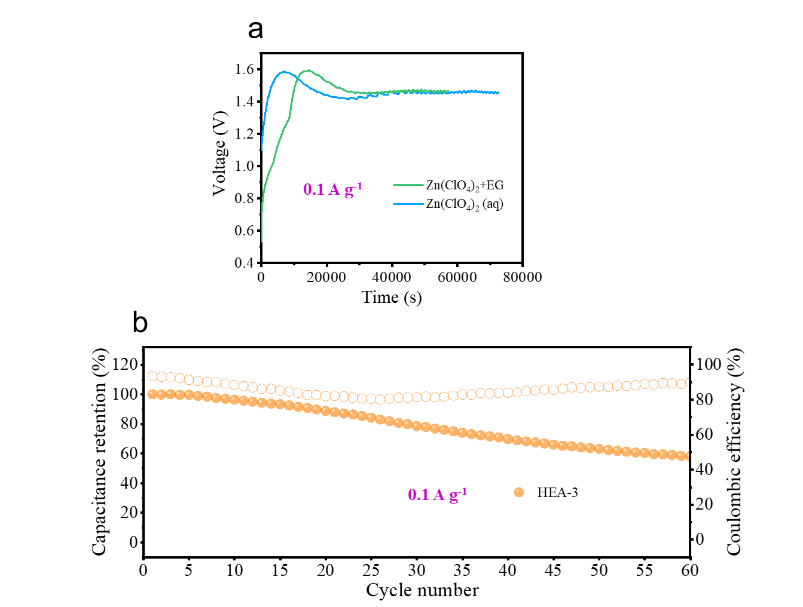


**Fig. S21** **a** GCD curves of Zn||PANI device with the Zn(ClO_4_)_2_ (aq) electrolyte and Zn(ClO_4_)_2_ (aq)+EG electrolyte at 0.1 A g^-1^ at room temperature. **b** Cycling performance of Zn||PANI device with HEA-3 electrolyte at 0.1 A g^-1^ at room temperature
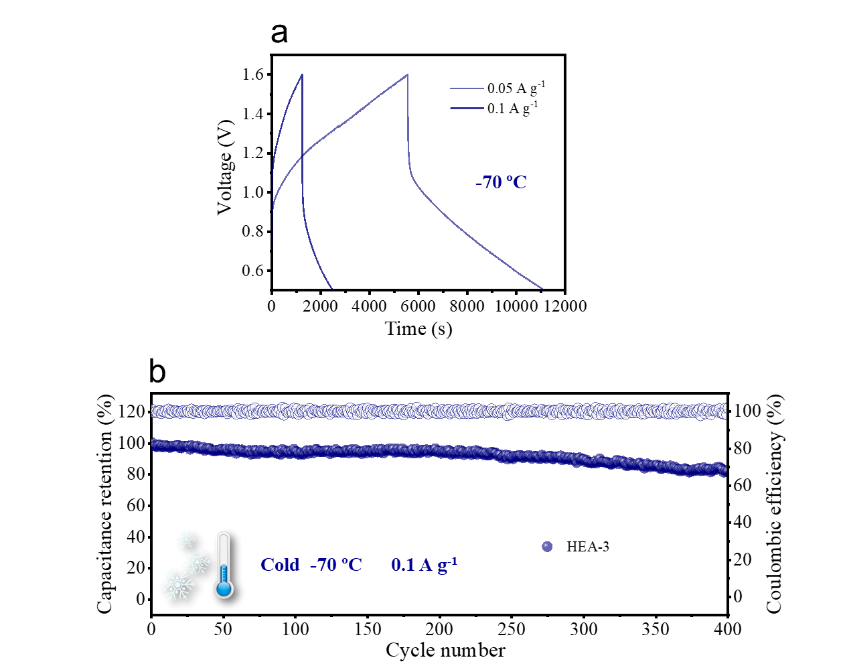


**Fig. S22** **a** GCD curves of Zn||PANI devices with different current densities at -70 ºC. **b** Cycling performance of Zn||PANI devices with a current density of 0.1 A g^-1^ at -70 ºC


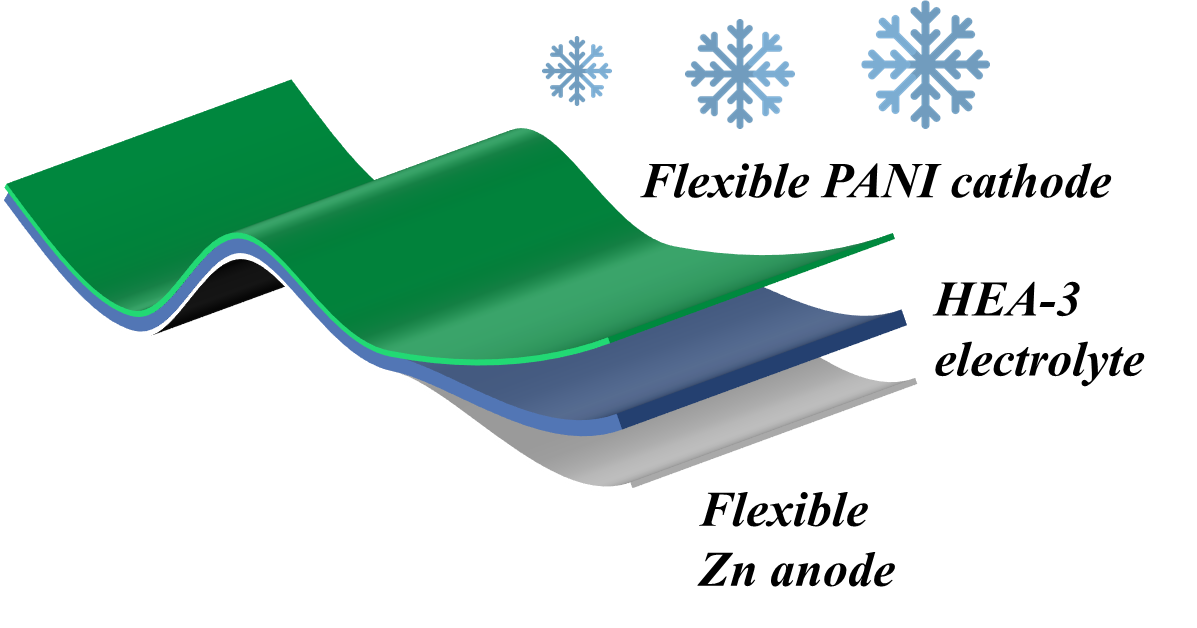


**Fig. S23** Schematic diagram of flexible Zn||PANI device

**
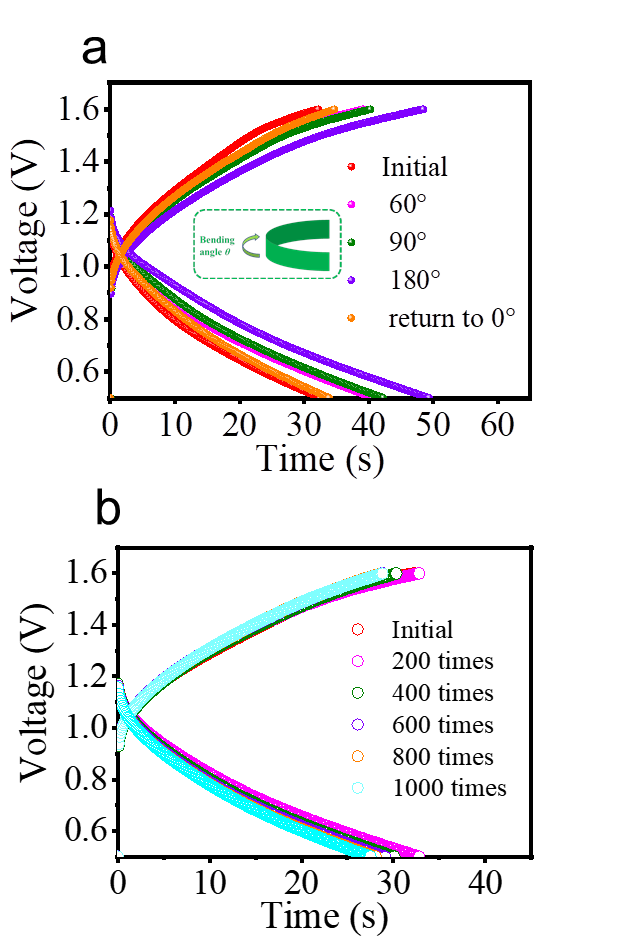
**

**Fig. S24** **a** GCD curves of flexible device under different bending conditions; **b** GCD curves of flexible device for various bending times at a bending angle of 180º

**Table S1** Comparison of low temperature performance of anti-freezing gel electrolyte and Zn-based devices

| System | electrolyte | Ionic conductivity /mS cm^-1^ | Low temperature performance | | | Refs |
| --- | --- | --- | --- | --- | --- | --- |
|  |  |  | capacity | Cyclig number | Working temperature (ºC) |  |
| Zn\|\|AC | PVA/MMT/  Zn(ClO_4_)_2_ | —— | 110 F g^-1^ | 10000 | -20 | [S17] |
| Zn\|\|AC | PAMPS/PAAm/EG/ZnCl_2_/NH_4_Cl | 1  (-30 ) | 48 F g^-1^ | 5500 | -30 | [S18] |
| AC\|\|δ-MnO_2_ | PVA/glycerol/  Zn(Cl)_2_ | 0.21  (-50 °C) | 8 mAh g^-1^ | 100 | -30 | [S2] |
| Zn\|\|δ-MnO_2_ | PAM/EG/ GO/ZnSO_4_/  MnSO_4_ | 14.9  (-20 °C ) | 183.2 mAh g^-1^ | 1000 | -20 | [S8] |
| Zn\|\|PANI | PAM/ZnCl_2_ | 9.93  (-20 °C) | 0.616 mAh cm^-2^ | 100 | -20 | [S7] |
| Zn\|\|PANI | PAMPS/PAM/EG/ZnCl_2_/NH_4_Cl | 1.62  (-30 ºC) | 59.7 mAh g^-1^ | 1500 | -30 | [S19] |
| Zn\|\|PANI | PVA/EG/ZnCl_2_/  NH_4_Cl | 2.89  (-30 ºC) | 58.8 mAh g^-1^ | 150 | -20 | [S20] |
| Zn\|\|PANI | PAM/CMCS/  Zn(ClO_4_)_2/_ | 7.8  (-30 °C) | 123 mAh g^-1^ | 2500 | -30 | [S21] |
| Zn\|\|Au-CNT-PANI | PAM/EG  Zn(OTf)_2_ | —— | 160.3 mAh g^-1^ | 600 | -20 | [S22] |
| Zn@CC\|\|GNP | PVA/EG/Zn(CF_3_SO_3_)_2_ | 5.6  (-30 °C) | 202.8 F g^-1^  (133.5 mAh g^-1^) | 30000 | -20 | [S3] |
| Zn@CC\|\|PANI | HEA-3 | 7.97  (-30 °C)  4.12  (-50 °C) | 244.8 F g^-1^/  (109.2 mAh g^-1^)  96.9 F g^-1^ | 30000  400 | -40  -70 | This work |

**Table S2** Comparison of the room temperature cycling stability of the reported Zn(ClO_4_)_2_-based electrolyte system

| Electrolyte system | Zn\|\|Zn cell  (current density/plating density) | Zn\|\|Zn cell Cycling life | DOD | Coulombic efficiency of half-cell | Refs |
| --- | --- | --- | --- | --- | --- |
| Sulfolane/  Zn(ClO_4_)_2_·6H_2_O | 0.2 mA cm^-2^/0.1 mAh cm^-2^  0.5 mA cm^-2^/0.5 mAh cm^-2^ | 1000 h  800 h | —— | 98% | [S23] |
| Succinonitrile/  Zn(ClO_4_)_2_·6H_2_O | 0.05 mA cm^-2^/0.5 mAh cm^-2^ | 800 h | —— | 98.4% | [S24] |
| NaClO_4_/H_2_O/  Zn(ClO_4_)_2_ | 0.2 mA cm^-2^/0.2 mAh cm^-2^ | 1000 h | —— | 98.2% | [S25] |
| Adiponitrile/H_2_O/Zn(ClO_4_)_2_ | 0.5 mA cm^-2^/0.5 mAh cm^-2^  1 mA cm^-2^/1 mAh cm^-2^ | 265 h  155 h | 0.85%  1.71% | 62.92% | [S26] |
| *β*-cyclodextrin/H_2_O/Zn(ClO_4_)_2_ | 1 mA cm^-2^/1 mAh cm^-2^  5 mA cm^-2^/5 mAh cm^-2^ | 1000 h  140h | 1.71%  30% | 97.6% | [S27] |
| NaClO_4_·H_2_O/H_2_O/Zn(ClO_4_)_2_·6H_2_O | 0.5 mA cm^-2^/0.25 mAh cm^-2^ | 3500 h | 0.43% | 98.3% | [S28] |
| HEA-3 | 1 mA cm^-2^/1 mAh cm^-2^  5 mA cm^-2^/5 mAh cm^-2^ | 1700 h  >110 h | 3.42%  17.1% | 99.4% | This work |

**Table S3** Comparison of low temperature performance of Zn||Zn cells with different electrolytes

| Electrolyte system | Current density  (mA cm^-2^) | Cumulative capacity  (mAh cm^-2^) | Temperature (ºC) | Refs |
| --- | --- | --- | --- | --- |
| 2 M ZnSO_4_ (40 vol%EG+60vol% H_2_O) | 2 | 160 | -20 | [S29] |
| 4 M Zn(BF_4_)_2_ (aq) | 0.5 | 750 | -30 | [S30] |
| GG/SA/EG | 0.2 | 40 | -20 | [S17] |
| 3M Zn(ClO_4_)_2_ (aq) | 0.5 | 200 | -30 | [S31] |
| CSAM-C | 1 | 1000 | -30 | [S5] |
| ZL-PAAm | 1 | 100 | -20 | [S32] |
| 2 M Zn(CF_3_SO_3_)_2_ (aq) | 0.2 | 80 | -30 | [S33] |
| Zn(CF_3_SO_3_)_2_/methanol (Me56) | 1 | 200 | -20 | [S34] |
| ZS/GL/AN | 0.2 | 100 | -20 | [S35] |
| HEA-3 | 1 | 1400 | -40 | This work |

**Supplementary References**

1. X. Zhu, C. Ji, Q. Meng, H. Mi, Q. Yang et al., Freeze-Tolerant Hydrogel Electrolyte with High Strength for Stable Operation of Flexible Zinc-Ion Hybrid Supercapacitors. Small **18**, (2022) 2200055. [https://doi.org/10.1002/smll.202200055](https://doi.org/https://doi.org/10.1002/smll.202200055)
2. Y. Jiang, K. Ma, M. Sun, Y. Li, J. Liu, All-Climate Stretchable Dendrite-Free Zn-Ion Hybrid Supercapacitors Enabled by Hydrogel Electrolyte Engineering. Energy & Environmental Materials **6**, (2023) e12357. [https://doi.org/10.1002/eem2.12357](https://doi.org/https://doi.org/10.1002/eem2.12357)
3. J. Liu, Z. Khanam, S. Ahmed, T. Wang, H. Wang, S. Song, Flexible Antifreeze Zn-Ion Hybrid Supercapacitor Based on Gel Electrolyte with Graphene Electrodes. ACS Appl. Mater. Interfaces **13**, 16454–16468 (2021). <https://doi.org/10.1021/acsami.1c02242>
4. Y. Wang, Y. Chen, A flexible zinc-ion battery based on the optimized concentrated hydrogel electrolyte for enhanced performance at subzero temperature. Electrochim. Acta **395**, 139178 (2021). <https://doi.org/10.1016/j.electacta.2021.139178>
5. J. Wang, Y. Huang, B. Liu, Z. Li, J. Zhang et al., Flexible and anti-freezing zinc-ion batteries using a guar-gum/sodium-alginate/ethylene-glycol hydrogel electrolyte, Energy Storage Materials **41**, 599-605 (2021). <https://doi.org/10.1016/j.ensm.2021.06.034>
6. S. Huang, L. Hou, T. Li, Y. Jiao, P. Wu, Antifreezing Hydrogel Electrolyte with Ternary Hydrogen Bonding for High-Performance Zinc-Ion Batteries, Adv. Mater. **34**, e2110140 (2022). <https://doi.org/10.1002/adma.202110140>
7. Y. Liu, X. Zhou, Y. Bai, R. Liu, X. Li et al., Engineering integrated structure for high-performance flexible zinc-ion batteries, Chemical Engineering Journal **417**, 127955 (2021). [https://doi.org/10.1016/j.cej.2020.127955](https://doi.org/https://doi.org/10.1016/j.cej.2020.127955)
8. Y. Quan, M. Chen, W. Zhou, Q. Tian, J. Chen, High-Performance Anti-freezing Flexible Zn-MnO_2_ Battery Based on Polyacrylamide/Graphene Oxide/Ethylene Glycol Gel Electrolyte, Frontiers in Chemistry **8**, 603 (2020). <https://doi.org/10.3389/fchem.2020.00603>
9. F. Mo, G. Liang, Q. Meng, Z. Liu, H. Li et al., A flexible rechargeable aqueous zinc manganese-dioxide battery working at −20 °C. Energ. Environ. Sci. **12**, 706-715 (2019). <https://doi.org/10.1039/C8EE02892C>
10. H. Wang, J. Liu, S. Ahmed, T. Wang, S. Song, Freeze-tolerant gel electrolyte membrane for flexible Zn-ion hybrid supercapacitor. Journal of Energy Storage **56**, 105923 (2022). [https://doi.org/10.1016/j.est.2022.105923](https://doi.org/https://doi.org/10.1016/j.est.2022.105923)
11. M. Qiu, H. Liu, B. Tawiah, H. Jia, S. Fu, Zwitterionic triple-network hydrogel electrolyte for advanced flexible zinc ion batteries. Composites Communications **28**, 100942 (2021). [https://doi.org/10.1016/j.coco.2021.100942](https://doi.org/https://doi.org/10.1016/j.coco.2021.100942)
12. Z. Liu, D. Wang, Z. Tang, G. Liang, Q. Yang et al., A mechanically durable and device-level tough Zn-MnO_2_ battery with high flexibility, Energy Storage Materials **23**, 636-645 (2019),.
13. B. Wang, J. Li, C. Hou, Q. Zhang, Y. Li, H. Wang, Stable Hydrogel Electrolytes for Flexible and Submarine-Use Zn-Ion Batteries, ACS Appl. Mater. Inter. **12**, 46005-46014 (2020). <https://doi.org/10.1021/acsami.0c12313>
14. C. Nusrath Unnisa, S. Chitra, S. Selvasekarapandian, S. Monisha, G. Nirmala Devi, V. Moniha et al., Development of poly(glycerol suberate) polyester (PGS)–PVA blend polymer electrolytes with NH4SCN and its application Ionics **24**, 1979-1993 (2018). <https://doi.org/10.1007/s11581-018-2466-x>
15. A.S. Samsudin, M.A. Saadiah, Ionic conduction study of enhanced amorphous solid bio-polymer electrolytes based carboxymethyl cellulose doped NH_4_Br, Journal of Non-Crystalline Solids **497**, 19-29 (2018). <https://doi.org/10.1016/j.jnoncrysol.2018.05.027>
16. L. Li, J. Wang, P. Yang, S. Guo, H. Wang et al., Preparation and characterization of gel polymer electrolytes containing N-butyl-N-methylpyrrolidinium bis(trifluoromethanesulfonyl) imide ionic liquid for lithium ion batteries, Electrochim. Acta **88**, 147-156 (2013). <https://doi.org/10.1016/j.electacta.2012.10.018>
17. G. Yang, J. Huang, X. Wan, Y. Zhu, B. Liu et al., A low cost, wide temperature range, and high energy density flexible quasi-solid-state zinc-ion hybrid supercapacitors enabled by sustainable cathode and electrolyte design Nano Energy **90**, 106500 (2021). [https://doi.org/10.1016/j.nanoen.2021.106500](https://doi.org/https://doi.org/10.1016/j.nanoen.2021.106500)
18. H. Wang, X. Li, D. Jiang, S. Wu, W. Yi, et al., Organohydrogel electrolyte-based flexible zinc-ion hybrid supercapacitors with dendrite-free anode, broad temperature adaptability and ultralong cycling life. J. Power Sources **528**, 231210 (2022). [https://doi.org/10.1016/j.jpowsour.2022.231210](https://doi.org/https://doi.org/10.1016/j.jpowsour.2022.231210)
19. X. Li, H. Wang, X. Sun, J. Li, Y.-N. Liu, Flexible Wide-Temperature Zinc-Ion Battery Enabled by an Ethylene Glycol-Based Organohydrogel Electrolyte. ACS Applied Energy Materials **4**, 12718-12727 (2021). <https://doi.org/10.1021/acsaem.1c02433>
20. Z. Cong, W. Guo, P. Zhang, W. Sha, Z. Guo, et al., Wearable Antifreezing Fiber-Shaped Zn/PANI Batteries with Suppressed Zn Dendrites and Operation in Sweat Electrolytes, Acs Appl. Mater. Inter. **13**, 17608-17617 (2021). <https://doi.org/10.1021/acsami.1c02065>
21. S. Huang, L. Hou, T. Li, Y. Jiao, P. Wu, Antifreezing Hydrogel Electrolyte with Ternary Hydrogen Bonding for High-Performance Zinc-Ion Batteries. Adv. Mater. **34**, 2110140 (2022). [https://doi.org/10.1002/adma.202110140](https://doi.org/https://doi.org/10.1002/adma.202110140)
22. X. Jin, L. Song, C. Dai, H. Ma, Y. Xiao et al., A self-healing zinc ion battery under -20 °C. Energy Storage Materials **44**, 517-526 (2022). [https://doi.org/10.1016/j.ensm.2021.11.004](https://doi.org/https://doi.org/10.1016/j.ensm.2021.11.004)
23. X. Lin, G. Zhou, M.J. Robson, J. Yu, S.C.T. Kwok et al., Hydrated Deep Eutectic Electrolytes for High-Performance Zn-Ion Batteries Capable of Low-Temperature Operation. Adv Funct Mater **32**, 2109322 (2022). <https://doi.org/10.1002/adfm.202109322>
24. W. Yang, X. Du, J. Zhao, Z. Chen, J. Li et al., Hydrated Eutectic Electrolytes with Ligand-Oriented Solvation Shells for Long-Cycling Zinc-Organic Batteries. Joule **4**, 1557-1574 (2020). <https://doi.org/10.1016/j.joule.2020.05.018>
25. Y. Zhu, J. Yin, X. Zheng, A.-H. Emwas, Y. Lei et al., Concentrated dual-cation electrolyte strategy for aqueous zinc-ion batteries. Energ Environ Sci **14**, 4463-4473 (2021). <https://doi.org/10.1039/D1EE01472B>
26. G. Liu, Y. Tang, Y. Wei, H. Li, J. Yan et al., Hydrophobic Ion Barrier-Enabled Ultradurable Zn (002) Plane Orientation towards Long-Life Anode-Less Zn Batteries. Angewandte Chemie International Edition **63**, e202407639 (2024). <https://doi.org/10.1002/anie.202407639>
27. M. Qiu, P. Sun, Y. Wang, L. Ma, C. Zhi et al., Anion-Trap Engineering toward Remarkable Crystallographic Reorientation and Efficient Cation Migration of Zn Ion Batteries. Angewandte Chemie International Edition **61**, e202210979 (2022). <https://doi.org/10.1002/anie.202210979>
28. Y.-S. Li, L.-S. Geng, B.-M. Zhang, Z.-H. Wei, H. Fan et al., Concentrated perchlorate-based electrolyte facilitates Zn anode-compatible in situ solid electrolyte interphase. Rare Metals **44**, 950-960 (2025). <https://doi.org/10.1007/s12598-024-02972-7>
29. N. Chang, T. Li, R. Li, S. Wang, Y. Yin, et al. An aqueous hybrid electrolyte for low-temperature zinc-based energy storage devices. Energ Environ Sci **13**, 3527-3535 (2020). <https://doi.org/10.1039/d0ee01538e>
30. T. Sun, X. Yuan, K. Wang, S. Zheng, J. Shi et al., An ultralow-temperature aqueous zinc-ion battery. J. Mater. Chem. A **9**, 7042-7047 (2021). <https://doi.org/10.1039/d0ta12409e>
31. S. Gao, B. Li, H. Tan, F. Xia, O. Dahunsi et al., High-Energy and Stable Subfreezing Aqueous Zn–MnO2 Batteries with Selective and Pseudocapacitive Zn-Ion Insertion in MnO_2_. Adv. Mater. **34**, 2201510 (2022). [https://doi.org/10.1002/adma.202201510](https://doi.org/https://doi.org/10.1002/adma.202201510)
32. M. Zhu, X. Wang, H. Tang, J. Wang, Q. Hao et al., Antifreezing Hydrogel with High Zinc Reversibility for Flexible and Durable Aqueous Batteries by Cooperative Hydrated Cations. Adv. Funct. Mater. **30**, 1907218 (2020). [https://doi.org/10.1002/adfm.201907218](https://doi.org/https://doi.org/10.1002/adfm.201907218)
33. Q. Zhang, K. Xia, Y. Ma, Y. Lu, L. Li et al., Chaotropic Anion and Fast-Kinetics Cathode Enabling Low-Temperature Aqueous Zn Batteries, ACS Energy Lett. **6**, 2704-2712 (2021). <https://doi.org/10.1021/acsenergylett.1c01054>
34. W. Xu, C. Liu, S. Ren, D. Lee, J. Gwon, et al., A cellulose nanofiber–polyacrylamide hydrogel based on a co-electrolyte system for solid-state zinc ion batteries to operate at extremely cold temperatures. J. Mater. Chem. A **9**, 25651-25662 (2021). <https://doi.org/10.1039/D1TA08023G>
35. T.T. Wei, Y.K. Ren, Z.Q. Li, X.X. Zhang, D.H. Ji et al., Bonding interaction regulation in hydrogel electrolyte enable dendrite-free aqueous zinc-ion batteries from -20 to 60 ºC. Chemical Engineering Journal **434**, 134646 (2022). <https://doi.org/10.1016/j.cej.2022.134646>
